# Anti-HIV and Antimicrobial Activity of 7-Hydrazino-8-hydroxyquinoline-Based Aromatic Hydrazones

**DOI:** 10.3390/ijms26178402

**Published:** 2025-08-29

**Authors:** Yaroslav V. Kozmenko, Marat M. Khisamov, Svetlana V. Revtovich, Sergey P. Korolev, Daria K. Sherman, Vasiliy V. Spiridonov, Lyudmila B. Kalnina, Vladimir T. Valuev-Elliston, Marina B. Gottikh, Sergey N. Kochetkov, Anastasia S. Zemskaya, Pavel N. Solyev

**Affiliations:** 1 Engelhardt Institute of Molecular Biology, Moscow 119991, Russia; yrko2002@gmail.com (Y.V.K.); hisamovmaratag@mail.ru (M.M.K.); svetla21@mail.ru (S.V.R.); dar.sher.man0@gmail.com (D.K.S.); gansfaust@mail.ru (V.T.V.-E.); snk1952@gmail.com (S.N.K.); a.zemskaia@mail.ru (A.S.Z.); 2 Chemistry Department, Lomonosov Moscow State University, Moscow 119992, Russia; spkorolev@mail.ru (S.P.K.); vasya_spiridonov@mail.ru (V.V.S.); 3 Belozersky Research Institute of Physico-Chemical Biology, Lomonosov Moscow State University, Moscow 119992, Russia; gottikh@belozersky.msu.ru; 4 Ivanovsky Institute of Virology, N. F. Gamaleya National Research Center of Epidemiology and Microbiology, Moscow 123098, Russia; klb3@yandex.ru

**Keywords:** 8-hydroxyquinoline, hydrazones, integrase, Ku, antibacterial activity, alginate

## Abstract

Aromatic hydrazones of 7-hydrazino-8-hydroxyquinoline were studied as anti-HIV and antibacterial compounds. A set of the compounds with different aromatic moieties bearing electron-donating and electron-withdrawing substituents has been selected and obtained via the hydrazo coupling and “one-pot” click reaction with aldehydes. The compounds possess activity against both bacterial and fungal targets. Cellular Ku70-inhibiting activity has been found for the series, opening a new class of inhibitors for potential anti-HIV treatment. The compounds display anti-HIV activity in infected cells at submicromolar concentrations. Their low solubility can be overcome by incorporation in water-soluble neutral polyalginate microgels (33% wt load of the compound).

## 1. Introduction

The demand for new drug candidates has significantly increased lately due to viral pandemics and threats of global antibiotic resistance to known antimicrobial drugs. Despite many techniques for the search of new drugs, like high-throughput screening (HTS), machine-learning-assisted iterative screening, microfluidics, and cytometry, the development of a hit compound still requires some luck and the use of a statistically relevant pharmacophore. Coincidently, research publications on 8-hydroxyquinoline (8-HQ) derivatives and their biological activity double every decade, which makes them a perfect starting point for drug screening towards new target inhibition in pathogens [1].

8-HQ is a versatile coordinative heterocycle frequently occurring in the structure of drugs and bioactive compounds, which is considered to be the “privileged structure” for application in medicinal chemistry, photochemistry, and agrochemistry. Thanks to its low price and cost-effective synthesis, non-substituted 8-HQ is still used as an antiseptic and antiprotozoal veterinary drug in many countries. Based on the 8-HQ core and 7-amino fragment in its structure, several fluoroquinolone antibiotics have been designed; among them, levofloxacin covered almost 30% of the fluoroquinolone antibiotics market in the 1991–2013 period [2]. However, microbial infections are getting out of clinical control, with multidrug resistance (MDR) emergence and dissemination of evolved resistant strains, requiring the search for a new generation of antimicrobials. An additional threat from MDR bacteria is posed to a highly susceptible group of immune-suppressed patients suffering from viral infections, who usually bear opportunistic microbial and viral co-infections within their disease. This situation can be regarded as a chance for a ubiquitous 8-HQ framework to serve as a multitargeted pharmacophore in drug design for new generations of anti-HIV agents, since many quinoline derivatives are found to represent integrase and Rev protein inhibitors [3].

**Scheme 1:**
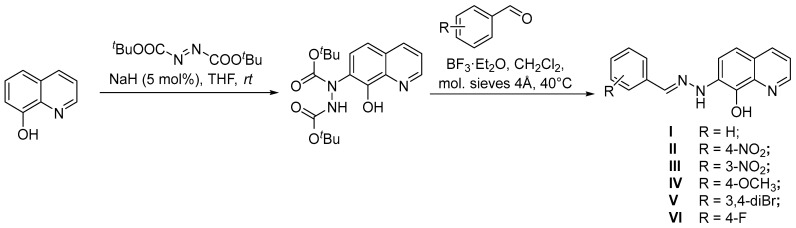
7-Hydrazo-8-hydroxyquinoline derivatives synthesis using hydrazo coupling and aldehyde click reaction.

Recently, we have discovered a new hydrazo coupling reaction, leading to products with potential biological activity (Scheme 1). In particular, a new, efficient method of 8-HQ functionalization with azodicarboxylate esters allows us to obtain 7-hydrazino-8-hydroxyquinolines using transition-metal-free basic conditions [4], complementing other known 8-HQ derivatization reactions; the Betty reaction [5]; esterification and etherification of the phenolic hydroxy group [6,7]; and electrophilic substitution reactions (mainly, halogenations) [8]. The proposed new method was also applied to the synthesis of 4-hydroxyphenylhydrazine and allowed the yield of this industrial precursor to be doubled. Access to the new class of 7-hydrazino-8-HQ derivatives could contribute to azo dyes and pigments, but their antiproliferative, antibacterial, and antiviral activities remain to be discovered. Hydrazone and oxime moieties are rarely used in drug design, but at the same time, they bring the necessary conformational rigidity to the structures and sufficient stability to hydrolysis when conjugated with the aromatic ring [9,10].

In the current study, we summarize some of the biological effects of aromatic hydrazones of 7-hydrazino-8-hydroxyquinoline. A set of compounds with different aromatic moieties with electron-donating and electron-withdrawing substituents has been selected and obtained via the hydrazo coupling and “one-pot” click reaction with aldehydes (Scheme 1). Their spectral data correspond to and are characteristic of those published previously [4].

## 2. Results and Discussion

### 2.1. Compounds Selection

**Scheme 2:**
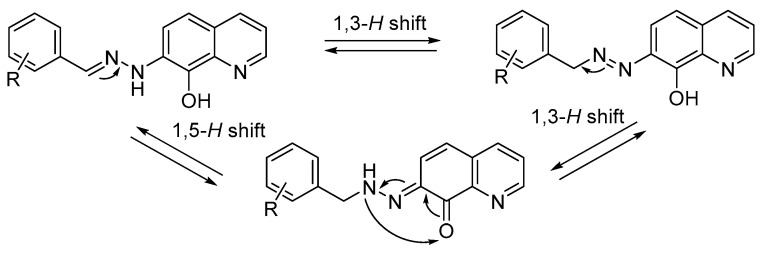
Azo-hydrazo tautomerism in hydrazones of 7-hydrazo-8-hydroxyquinoline.

Choosing the right structure in drug design is a strategic but contentious issue. 8-HQ core frequently occurs in many natural products and serves as a basis for hundreds of drug candidates [11]. It is quite common to find a bicyclic motif attached to another benzene ring via a linker in antiviral and antibiotic compounds. 7-Hydrazino derivative of 8-HQ gains even more chelating properties towards cations than the starting 8-HQ, giving possibilities for binding in the active sites of viral enzymes and offering a simple derivatization into hydrazones. The characteristic feature of this structure is determined by the resonance-stabilized conjugated flat π-system with the predominant *anti*-orientation of the hydrazone linkage. Since the conjugated flat π-system possesses chromophore groups that provide absorption and emission in the UV and visible ranges, hydrazones display exceptional solvatochromism, depending on the solvent’s nature, the mesomeric effects of the aromatic substituents, pH, and the compound’s concentration (Figure S29). An azo-fragment with a mobile hydrogen atom experiences azo-hydrazone tautomerism (Scheme 2). The +M effect of substituents in the ring stabilizes the benzylidene imine fragment of the molecule and hinders the transition to the quinone hydrazone form. At the same time, this tautomerism drastically lowers the water solubility of the compounds, broadens signals in NMR spectra and sometimes even adds up signals of other tautomeric forms. The same phenomena can be observed when comparing salicylic acid (solubility 2.2 mg/mL) with *p*-hydroxybenzoic (solubility 5 mg/mL), *m*-hydroxybenzoic (solubility 7.25 mg/mL), benzoic (3.44 mg/mL) acids, or phenol (>80 mg/mL) [12]. Nevertheless, the flat structure of the hydrazone, which is gained through tautomerism, is much appreciated in the design of anti-HIV drug candidates.

Aromatic substituents for hydrazones have been selected to gain more diversity in biological action against several targets, according to the general expectations of different electronic effects and knowledge of the previously found active moieties. Therefore, as a basis, the non-substituted benzylidene derivative (**I**) would represent the general effects of the aromatic core. Like nitrofurans, nitro derivatives (**II** and **III**) could serve as substrates for bacterial nitroreductase, leading to NO-triggered DNA and protein damage in pathogens. Alkoxy (**IV**) and halogen (**V** and **VI**) fragments have been selected for their general presence in the structure of antiviral protein inhibitors (in particular, integrase and Rev protein inhibitors), as well as in a few representative examples of Ku70 protein inhibitors [13,14,15,16,17]. The bromine-substituted derivative (**V**) is capable of additional halogen bonding, which may also affect binding in the protein’s active site. The fluorobenzylidene product (**VI**), coupled with highly coordinative 7-hydrazino-8HQ, may provide additional antimicrobial properties, serving as a fluconazole mimetic. Thus, the series of electron-donating (**IV**) and electron-withdrawing (**II**, **III**, **V**, **VI**) substituents with different mesomeric effects has been utilized, with the aim of fulfilling the desired activity, in as many drug targets in bacteria and HIV as possible, with this structural type.

### 2.2. Anti-HIV Activity

**Figure 1:**
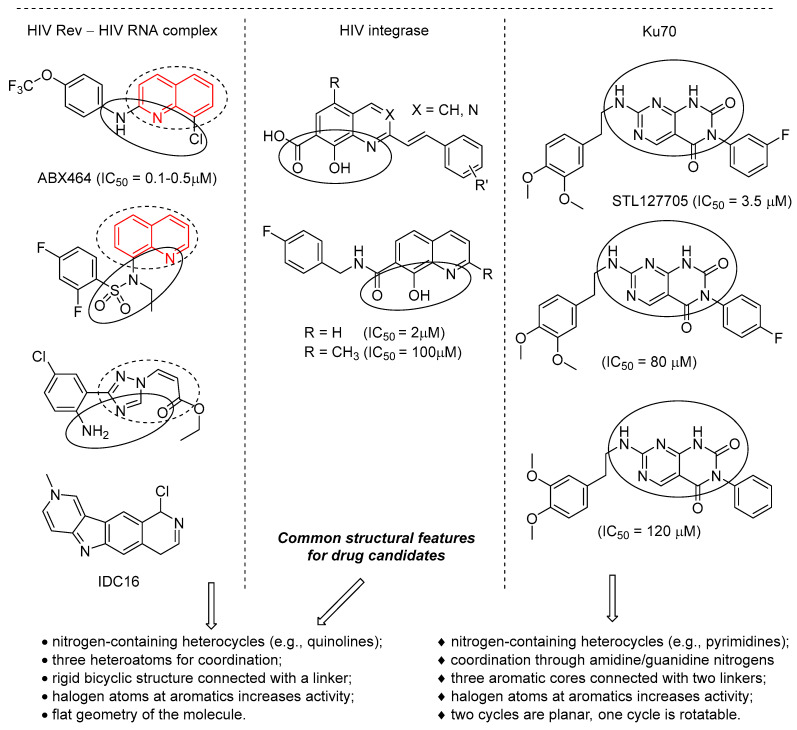
Common structural motifs and distinctive features in the previously reported compounds targeting HIV Rev [15,16], HIV integrase [13,14], and Ku70 [17] for potential anti-HIV therapy.

We started our evaluation of the biological properties of 7-hydrazino-8-HQ hydrazones with anti-HIV targets, since the obtained compounds possess similarities in drug design with many anti-HIV compounds [13]. For example, they fit the basic geometry of the HIV integrase inhibitors previously developed by other research groups (Figure 1). Most quinoline-containing HIV integrase inhibitors are revised analogues of diketo acids with lower toxicity [14]. The usage of 8-HQ-functionalized fragments is determined by its strong coordination with two magnesium ions in the integrase active site, that are also locked by an additional (usually electron-donating or mesomerically stabilized) heteroatom in the nearby linker/substituent. At the same time, the second aromatic residue on the linker and the linker itself are important motifs for enhanced binding with the enzyme. Many potential HIV Rev protein inhibitors share similarity in the design with integrase inhibitors: a nitrogen heterocycle-coordinating scaffold and a substituted (preferably with halogens) aromatic substituent adjacent via a short linker [13,14,15]. Both proteins are prone to bind with flat or polyaromatic molecules, in contrast to non-nucleoside reverse transcriptase inhibitors that require three aromatic cycles linked together. In addition, inhibitors of human Ku protein, which functions both in mammals (where it can be auxiliary) and in pathogens (likely to be essential for viral replication), may require relatively close geometry with a broader linker length, but structural data on these inhibitors are still scarce.

Thus, all these targets can be tested to determine the anti-HIV activity of the synthesized aromatic 7-hydrazino-8-hydroxyquinoline hydrazones.

#### 2.2.1. Ability to Disrupt Formation of HIV Rev–RNA Complex

In 2015, the first-in-class anti-HIV drug–candidate ABX464 was proposed, selectively inhibiting the functioning of a novel viral target—the Rev protein [18]. According to the preliminary data, ABX464 possessed a unique mechanism of action, disrupting an important function of the Rev protein binding with viral RNA via the Rev response element (RRE) motifs, thus preventing the replicated RNA from being transported from the cell nucleus to the cytoplasm and blocking the formation of proviral particles. Preclinical studies described the absence of mutant viral strains during ABX464 test treatment [18]. The compound was promptly introduced to clinical trials as a candidate for the anti-HIV drug and accomplished Phase IIa and Phase IIb; however, after 2020, it was repurposed as an experimental candidate against SARS-CoV-2 (ClinicalTrials.gov ID NCT04393038). However, once it founded the “golden rush” search for hit compounds against the new promising drug target, HIV Rev, clinical trials of ABX464 quickly changed its direction. ABX464 later entered Phase III as a drug candidate (obefazimod) for the treatment of moderate–severe active ulcerative colitis and Crohn’s disease (ClinicalTrials.gov ID NCT06456593).

A number of molecules were reported as Rev ligands; however, only a couple of examples were tested in cell cultures [15,16]. Rev binding with the synthetic ligand does not necessarily the block protein’s capability to bind with viral RNA or disrupt its functioning. Hence, the HIV-infected cell culture assays are essential for proper evaluation.

To explore the effect of the hydrazones **I**–**VI** on the Rev protein function we designed our own test system for screening different compounds for Rev–RRE complex disruption [19] and we checked 7-hydrazino-8-hydroxyquinoline derivatives. Unfortunately, none of these hydrazones possessed inhibitory activity at concentrations up to 100 μM (EMSA gels are represented in Figure S26).

#### 2.2.2. Activity Against HIV Integrase

Integrase carries out one of the key stages of the virus replication cycle—the insertion of a DNA copy of the genomic RNA into the genome of infected human cells. 7-Hydrazo-8HQ derivatives were checked for the possible inhibition of two integrase-catalyzed processes: as chain transfer inhibitors and as substrates for the catalytic domain. HIV integrase functions in two stages: first, having bound with viral DNA, the enzyme catalyzes the 3′-processing reaction, as a result of which the GT dinucleotide is removed from the 3′ end of each strand; at the second step, integrase catalyzes the strand transfer reaction responsible for the viral DNA incorporation into the host cell DNA. Here, 3′-processing inhibitors bind to the active site of integrase and prevent it from binding with viral DNA. However, most of these inhibitors statistically turn out to be inactive in vivo.

Hydrazones **I**–**VI** were found to inhibit strand transfer but not 3′-processing. Among the series, there is only one successful example—the 4-fluoro derivative **VI** with activity at a concentration of about 40 µM (Table 1). In addition, the three other derivatives (**I**, **III** and **IV**) showed weak activity, the remaining derivatives (**II** and **V**) were ineffective, compared to other known drug candidates (Figure S25). The strand transfer inhibitor L-708,906 [20] was used as a positive control compound, since it is known to inhibit both strand transfer (IC_50_ = 0.5 ± 0.1 μM) and 3′-processing (IC_50_ = 4.2 ± 1.6 μM). Thus, the hydrazone derivative with the fluorophenyl moiety **VI** binds to the integrase complex with viral DNA and prevents its binding to cellular DNA. Presumably, hydrazones chelate the magnesium ions in the active site of integrase.

#### 2.2.3. Ability to Disrupt Formation of Ku70/Ku80–DNA Complex

Some proteins of mammalian cells can potentially serve as a backdoor target for antiviral therapy. For example, such proteins operate multiple auxiliary functions in cells or their role can be duplicated by other proteins; however, their inhibition in infected cells influence life cycle of the virus in a profound manner. One example is the human Ku protein—a DNA repair heterodimer protein consisting of two subunits, Ku70 and Ku80. Ku protein binds DNA double-strand breaks through non-homologous end joining [21]; it is also involved in V(D)J recombination, transposable element-mediated rearrangements in the genome, telomere length maintenance, apoptosis, and regulation of ubiquitination [22]. Importantly, beyond cellular function, Ku70 is involved in the viral genes’ transcription in HIV due to its ability to bind with DNA [23]. Inhibitors of the Ku protein’s interaction with DNA are promising tools in the study of HIV transcription, as well as being potentially useful in antiviral and anticancer drugs. The relevance of Ku70 as a new drug target is supported by the reports about its indirect effects on other viral pathogens, including hepatitis B [24] and hepatitis C viruses [25]. Currently, only a few active inhibitors of Ku70 interaction with DNA have been identified and described, for example, STL127705 (IC_50_ = 3.5 μM) [17] and KuINins (IC_50_ = 12 μM on Ku/In complex inhibition at the stage of post-integrational DNA repair) [26], but no approved standard inhibitor with exceptional activity has been established yet (Figure 1).

We carried out three consecutive rounds of EMSA screening on recombinant Ku70 proteins with isotopically labelled DNA; we identified that 7-hydrazo-8HQ hydrazones are a new structural class of Ku/DNA interaction inhibitors with varying IC_50_ from 25 to 80 μM (Table 2). 3,4-Dibromobenzylidene performs the best among the series. Presumably, this class can allosterically impair the basic function of the Ku heterodimer.

#### 2.2.4. Antigen p24 Assay in HIV-Infected MT-4 Cells

**Figure 2:**
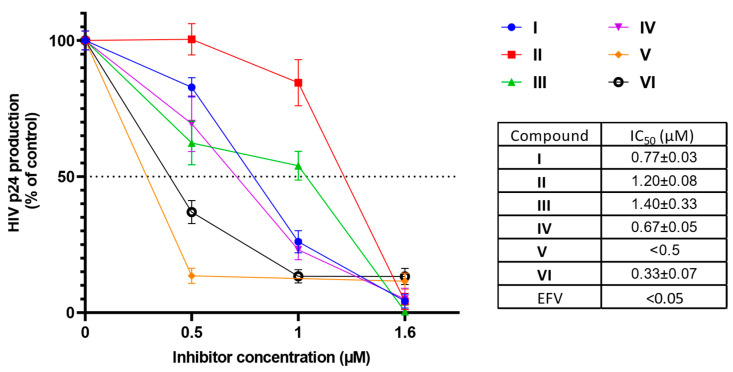
Activity of compounds against human immunodeficiency virus (HIV-1) in MT-4 infected cells.

To explore the overall anti-HIV action of the products, we performed an antigen p24 assay screening in the macrophage cell line MT-4, infected with HIV-1 (subtype B) in a dose-dependent manner. The non-nucleoside HIV reverse transcriptase inhibitor Efavirenz (EFV) with IC_50_ *<* 0.05 μM was used as a positive control (Figure 2). The tested aliquots were freshly prepared and applied in antigen p24 assay, but the compounds suffered from poor solubility in the cell medium in long-term experiments that caused self-aggregation at high concentrations after one day of incubation. Despite this, the hydrazones demonstrated profound activity in micromolar and submicromolar concentrations.

The compounds effectively prevented MT-4 cells from reaching the virus-induced syncytia formation at low micromolar concentrations (Figure S27).

### 2.3. Antimicrobial Activity

Quinoline antimicrobial compounds are widely represented by fluoroquinolone antibiotics based on the 3-carboxy-6-fluoro-4-quinolone pharmacophore; meanwhile, research on other quinoline-based derivatives has not achieved much success in attaining antibacterial or antifungal drug approvals. Up till now, the antimicrobial drug design of these compounds has relied on a molecular hybridization approach and chemical modifications; thus, it has remained underdeveloped. Therefore, to broaden the target scope, we tested our series of 7-hydrazo-8-HQ derivatives for antibacterial activity against representative Gram(+), Gram(–) bacteria, and fungus (Table 3). In the range of concentrations studied, the compounds were not effective against the Gram(–) bacterium *P. aeruginosa*; three out of six compounds—**II**, **III,** and **V**—inhibited the growth of Gram(+) bacterium *S. aureus* at MICs (minimal inhibitory concentrations) of 45–90 μM. Furthermore, all compounds had a pronounced toxic effect on the *C. albicans* fungus at MICs of 13–40 μM. The hit compound appeared to be nitro-derivative **II,** which showed the best antifungal (MIC = 13 μM) and antibacterial (MIC = 45.4 μM) efficacies.

### 2.4. Cytotoxicity

The toxicity of the compounds has been assessed by three independent assays: MTT, NR, and SRB. Presuming that the tested compounds may have an effect on mitochondria, we have broadened the common MTT assay with NR and SRB assays that assess the activity of lysosomes and the total protein content, respectively. This hypothesis was partially confirmed during our study (Table 4). The toxicity of the compounds correlates well either between the data obtained with NR and SRB, or MTT and SRB, but not between MTT and NR or all three methods. We assume that the SRB assay is the most suitable one for the study of 7-hydrazo-8-HQ derivatives, since this method does not depend on the compounds’ effects on cellular organelles. It is likely that compounds’ cytotoxicity profiles are affected by their low solubility in water and the cell culture medium.

Poor solubility is one of the main obstacles faced in the implementation of **I**–**VI** in cell-based biological studies. While raising DMSO percentage is not an option for cell culture experiments because of the increasing cytotoxic effects, there are alternative methods for delivering certain substances, such as liposomal nanosystems or guest–host complexes in cyclodextrins. However, the percentage of the incorporated substance rarely exceeds 10–12% using the above-mentioned approaches. One possible solution is to use a non-toxic carrier for drug delivery, for example, through the use of polymer microgels. These are three-dimensional cross-linked networks composed of hydrophilic polymers with a high water content. We have chosen sodium alginate as a basis for the polymer carrier, implying its biocompatibility with cell cultures.

Alginate is a block copolymer of l-glucuronate (G) and d-mannuronate (M), with the varying G:M ratio depending on its source. Being a natural and biodegradable polymer, it is safe for the human body. Currently, more than 200 different alginates with individual contents of G and M blocks are commercially available. It is considered that the G block participates in intermolecular crosslinking with divalent cations with high affinity for complex formation (Cu^2+^, Ca^2+^, Fe^2+^, etc.), with the formation of microgels. Mechanical properties, such as rigidity, density, and viscosity, are regulated by the G:M ratio and the type of cross-linking used, in addition to their molecular weights. The maximum viscosity is reached at pH values of 3–3.5 due to the protonation of the carboxylate groups and the formation of hydrogen bonds; but highly viscous microgels are usually undesirable, since cellular components could be damaged during cell culture assay incubation; hence, they are usually quenched or dried to the neutral pH. We have used regular G:M (1:1) alginate cross-linked with Cu^2+^ ions, which is the most common microgel formulation used for the incorporation of drugs. Microgels are often biocompatible, being structurally similar to the components of macromolecules in the body. In our tests, the alginate microgel without hydrazones did not influence cell proliferation in any of the three cytotoxicity assays.

Our preliminary studies have shown that hydrazones can be incorporated into the microgel with a good net load of 30% (assessed using ^1^H NMR, UV–Vis, and IR spectra; Figures S28 and S30–S32, Table S1) and gain a water solubility improvement of 200–250% (~20 mg in 1 mL of water for **I** incorporated in alginate (28.6% net load) vs. ~2.3 mg in 1 mL of water for **I**); the obtained microgels remain stable for several days, while non-incorporated hydrazones precipitate in aqueous solutions within half an hour.

As a result, microgel formulation can serve as a promising approach for gaining better water solubility for compounds like 7-hydrazino-8-HQ hydrazones. Their cytotoxic properties in cells were almost identical to those of the non-incorporated hydrazones (tested for **I** and calculated based on equivalent hydrazone quantity, according to the weight load in the polymer). Higher solubility of the incorporated forms eliminated plausible errors associated with the precipitation of substances in cellular assays.

## 3. Materials and Methods

All the starting materials (unless otherwise stated) were of reagent grade and higher purity, purchased from Acros Organics (Geel, Belgium) and Sigma-Aldrich (Burlington, MA, USA) and used without additional purification. Solvents were dried and purified according to standard procedures; LC-MS grade methanol was purchased from PanReac (Barcelona, Spain) and used without further purification. TLC was performed on Merck Kieselgel 60F_254_ (Darmstadt, Germany) with UV visualization. Column chromatography was performed on silica gel 40–63 μm from Merck (Darmstadt, Germany). ^1^H and ^13^C NMR spectra (δ, ppm; *J*, Hz) were registered on an AMX III 300 spectrometer (Bruker BioSpin GmbH, Karlsruhe, Germany) with a working frequency of 300 MHz for ^1^H NMR (Me_4_Si as an internal standard for organic solvents), 75 MHz for ^13^C NMR (with carbon–proton interaction decoupling) and on an Avance II 600 spectrometer (Bruker BioSpin GmbH, Karlsruhe, Germany) with a working frequency of 600 MHz for ^1^H NMR (Me_4_Si as an internal standard for organic solvents) and 151 MHz for ^13^C NMR (with carbon–proton interaction decoupling). High-resolution mass spectra (HRMS) were registered on a micrOTOF-Q II hybrid quadrupole time-of-flight mass spectrometer (Bruker Daltonics GmbH, Bremen, Germany) using electrospray ionization (ESI); the measurements were carried out in positive ion mode. The samples were injected into the mass spectrometer chamber using a syringe injection or from the Agilent 1260 HPLC system (Agilent Technologies, Santa Clara, CA, USA) equipped with an Agilent Poroshell 120 EC-C18 column (3.0 × 50 mm; 2.7 μm) and an identically packed security guard using an autosampler. The samples were in 50% methanol in water (MilliQ ultrapure water; Merck Millipore KGaA, Darmstadt, Germany). The column was eluted with a gradient of methanol (A) concentrations in water (B) with a flow rate of 400 μL/min in the following gradient parameters: 0–15% A for 6 min, 15–85% A for 1.5 min, 85–0% A for 0.1 min, and 0% A for 2.4 min. UV spectra were recorded on a Cary-50 spectrophotometer (Varian, Palo Alto, CA, USA), a Tecan Spark Multimode Microplate Reader (Tecan Group Ltd., Männedorf, Switzerland), and an iMark Microplate Reader (Bio-Rad, Hercules, CA, USA). IR Spectra were registered from a pressed KBr pellets with a Perkin-Elmer Paragon 1000 FT-IR spectrometer (Perkin-Elmer, Waltham, MA, USA) in a wavelength range 700–4000 cm^−1^. Radiolabeled RNA concentration was determined using liquid scintillation analyzer Tri-Carb 2810 (PerkinElmer, Waltham, MA, USA).

### 3.1. Chemical Synthesis

Starting di-*tert*-butyl 1-(8-hydroxyquinolin-7-yl)hydrazinyl-1,2-dicarboxylate was synthesized according to the previously described procedure and isolated as a beige solid (yield: 99%) [4]. Hydrazones **I**–**VI** were synthesized according to the previously described procedure and isolated as dark purple solids (yields: 50–90%) in *anti*-/*syn*-hydrazones mixture with the *anti*-isomer being the major one (60–90%) [4]. HPLC-HRMS, UV, and NMR spectra profiles are displayed in the Supplementary Information file (Figures S1–S24 and S28).

#### 3.1.1. 7-(2-Benzylidenohydrazinyl)-8-hydroxyquinoline (**I**) (*anti*-isomer)

^1^H NMR (CD_3_OD, δ): 9.38 (m, 1H, 2), 8.90 (m, 1H, 4), 8.06 (s, 1H, CH=N), 7.92 (m, 1H, 3), 7.65 (m, 3H, 6 + *o*-Ph), 7.43–7.30 (m, 4H, 5 + *m*-Ph + *p*-Ph). ^13^C NMR (CD_3_OD, δ): 144.09 (s, C4), 142.58 (s, C, CH=N), 142.38 (s, C2), 141.46 (s, C8), 136.72 (s, C9), 136.34 (s, *i*-Ph), 130.23 (s, C10), 129.91 (s, *p*-Ph), 129.69 (s, *o*-Ph), 127.37 (s, *m*-Ph), 120.72 (s, C3), 120.53 (s, C7), 119.53 (s, C5), 112.58 (s, C6). HRMS of C_16_H_13_N_3_O (*m*/*z*): calculated for [M + H]^+^ 264.1131, found 264.1122.

#### 3.1.2. 7-(4-Nitrobenzylidenohydrazinyl)-8-hydroxyquinoline (**II**) (*anti*-isomer)

^1^H NMR (CD_3_OD, δ): 9.42 (d, ^3^*J* 8.63 Hz, 1H, 2), 8.99 (d, ^3^*J* 5.22 Hz, 1H, 4), 8.24 (d, ^3^*J* 8.85 Hz, 2H, *m*-Ph), 8.16 (s, 1H, CH=N), 8.01 (dd, ^3^*J* 8.63 Hz, ^4^*J* 5.22 Hz, 1H, 3), 7.90 (d, ^3^*J* 8.85 Hz, 2H, *m*-Ph), 7.83–7.76 (m, 1H, 6), 7.49–7.47 (m, 1H, 5). ^3^C NMR (CD_3_OD, δ): 147.25 (s, C-NO_2_), 143.01 (s, C4), 141.84 (s, C8), 141.00 (C2), 137.60 (s, CH=N), 133.92 (s, C9), 130.55 (s, C10), 128.95 (s, *i*-Ph), 126.31 (s, *o*-Ph), 123.58 (s, *m*-Ph), 119.79 (s, C3), 119.34 (s, C7), 117.77 (s, C5), 112.12 (s, C6). HRMS of C_16_H_12_N_4_O_3_ (*m*/*z*): calculated for [M + H]^+^ 309.0982, found 309.0973.

#### 3.1.3. 7-(3-Nitrobenzylidenohydrazinyl)-8-hydroxyquinoline (**III**) (*anti*-isomer)

^1^H NMR (CD_3_OD, δ): 9.38 (d, ^3^*J* 11.21 Hz, 1H, 2), 8.94 (d, ^3^*J* 1.36, 1H, 4), 8.41 (s, 1H, 3), 8.11 (s, 1H, CH=N), 8.10 (dd, ^3^*J* 1.5 Hz, ^4^*J* 0.67 Hz, 1H, 4-Ph), 8.00–7.96 (m, 2H, 2-Ph + 6-Ph), 7.70 (d, ^3^*J* 8.63 Hz, 1H, 6), 7.59 (t, ^3^*J* 7.93 Hz, 5-Ph), 7.45 (d, ^3^*J* 8.63 Hz, 1H, 5). ^13^C NMR (CD_3_OD, δ): 148.64 (s, C-NO_2_), 142.67 (s, C4), 141.48 (s, C2), 140.49 (s, C8), 137.96 (s, CH=N), 137.34 (s, C9), 134.22 (s, C10), 131.54 (s, 6-Ph), 129.60 (s, 5-Ph), 128.36 (s, 1-Ph), 122.42 (s, 4-Ph), 119.85 (s, C3), 119.65 (s, 2-Ph), 119.23 (s, C7), 118.23 (s, C5), 111.85 (s, C6). HRMS of C_16_H_12_N_4_O_3_ (*m*/*z*): calculated for [M + H]^+^ 309.0982, found 309.0972.

#### 3.1.4. 7-(4-Methoxybenzylidenohydrazinyl)-8-hydroxyquinoline (**IV**) (*anti*-isomer)

^1^H NMR (CD_3_OD, δ): 9.39 (dd, ^3^*J* 8.62 Hz, ^4^*J* 1.36 Hz, 1H, 2), 8.90 (dd, ^3^*J* 5.45 Hz, ^4^*J* 1.36 Hz, 1H, 4), 8.01 (s, 1H, CH=N), 7.92 (dd, ^3^*J* 8.63 Hz, ^4^*J* 5.22Hz, 1H, 3), 7.63 (d, ^3^*J* 8.63 Hz, 1H, 6), 7.59–7.57 (m, 2H, *o*-Ph), 7.43 (d, ^3^*J* 8,63 Hz, 1H, 5), 6.93 (m, 2H, *m*-Ph), 3.83 (s, 3H, OCH_3_). ^13^C NMR (CD_3_OD, δ): 160.58 (s, *p*-Ph), 142.38 (s, C4), 141.65 (s, C2), 141.30 (s, CH=N), 139.40 (s, C8), 135.37 (s, C9), 128.41 (s, C10), 127.93 (s, C7), 127.46 (s, *o*-Ph), 119.10 (s, C3), 118.62 (s, C5), 113.77 (s, *m*-Ph), 110.86 (s, C6), 54.42 (s, OCH_3_). HRMS of C_17_H_15_N_3_O_2_ (*m*/*z*): calculated for [M + H]^+^ 294.1237, found 294.1230.

#### 3.1.5. 7-(3,4-Dibromobenzylidenohydrazinyl)-8-hydroxyquinoline (**V**) (*anti*-isomer)

^1^H NMR (CD_3_OD, δ): 9.26 (dd, ^3^*J* 8.63 Hz, ^4^*J* 1.23 Hz, 1H, 2), 8.90 (dd, ^3^*J* 5.33 Hz, ^4^*J* 1.21 Hz, 1H, 4), 7.91 (dd, ^3^*J* 8.63 Hz, ^4^*J* 5.34 Hz, 1H, 3), 7.84 (s, 1H, CH=N), 7.72 (d, ^3^*J* 1.88 Hz, 1H, 2-Ph), 7.58 (d, ^3^*J* 8.63 Hz, 1H, 6), 7.50 (d, ^3^*J* 8.63 Hz, 1H ,5-Ph), 7.39 (d, ^3^*J* 8.65 Hz, 2H, 6-Ph + 5). ^13^C NMR (CD_3_OD, δ): 142.48 (s, C4), 141.26 (s, C2), 140.19 (s, C8), 137.70 (s, CH=N), 136.45 (s, 1-Ph), 134.18 (s, C9), 133.48 (s, 5-Ph), 130.25 (s, 1-Ph), 128.24 (s, C10), 125.90 (s, 6-Ph), 124.33 (s, 3-Ph), 123.55 (s, 5-Ph), 119.48 (s, C3), 118.86 (s, C7), 118.31 (s, C5), 111.41 (s, C6). HRMS of C_16_H_11_Br_2_N_3_O (*m*/*z*): calculated for [M + H]^+^ 419.9342, found 419.9326;

#### 3.1.6. 7-(4-Fluorobenzylidenohydrazinyl)-8-hydroxyquinoline (**VI**) (*anti*-isomer)

^1^H NMR (CD_3_OD, δ): 8.82 (m, 1H, 2), 8.05 (m, 1H, 4), 7.68–7.44 (m, 5H, CH=N + 3 + 6 + *o*- Ph), 7.23–7.09 (m, 3H, *m*-Ph, 5). ^13^C NMR (CD_3_OD, δ): 164.30 (d, ^1^*J* 247.08, C-F), 146.85 (s, C2), 139.84 (s, C4), 136.57 (s, C9), 135.38 (s, *i*-Ph), 133.62 (s, C10), 128.97 (d, ^2^*J* 7.75 Hz, *o*-Ph), 121.05(s, C3), 116.50 (d, ^3^*J* 36.25 Hz, *m*-Ph), 115.54 (s, C5), 111.75 (s, C6). HRMS of C_16_H_12_FN_3_O (*m*/*z*): calculated for [M + H]^+^ 282.1037, found 282.1037.

### 3.2. Compound Testing

Compounds were synthesized according to the previously described protocols [4]. Stock solutions were diluted in 1% DMSO (BioReagent for molecular biology grade, Sigma-Aldrich, Burlington, MA, USA) for protein assays and antimicrobial testing, and in 1% ethanol (BioReagent for molecular biology grade, Sigma-Aldrich, Burlington, MA, USA) for cell culture assays.

### 3.3. Screening for HIV Integrase Activity

The recombinant HIV-1 Integrase (In) was isolated from the Rosetta *Escherichia coli* producer strain and purified without the addition of a detergent as described previously [27]. Radioactive ^32^P label was introduced at the 5′-end of the oligonucleotide. Testing of the compounds was performed according to the previously published procedure [24]. Briefly: 5 nM ^32^P-labeled 21 mer DNA duplex (5′-GTGTGGAAAATCTCTAGCAGT-3′ and 5′-ACTGCTAGAGATTTTCACAC-3′) for 3′-processing reaction or 10 nM 19/21 mer DNA duplex (5′-GTGTGGAAAATCTCTAGCA-3′ and 5′-ACTGCTAGAGATTTTCACAC-3′) for strand transfer was incubated in 20 µL of the buffer (20 mM HEPES, pH 7.2, 7.5 mM MgCl_2_, 1 mM DTT) in the presence of 100 nM integrase and increasing concentrations of the inhibitor at 37 °C for 2 h. The reaction products were assayed by 20% polyacrylamide gel electrophoresis (PAGE) with 7 M urea. The result was visualized by radioautography on a Typhoon FLA 9500 PhosphorImager (GE Healthcare Technologies, Chicago, IL, USA). The counting of radioautograms was performed using QuantityOne 4.6.6 software (Bio-Rad, Hercules, CA, USA). Calculation of IC_50_ parameters was carried out by Prism 8.0.1 software (GraphPad Software, San-Diego, CA, USA).

### 3.4. Ku Protein Assay

A bicistronic construct was used for the simultaneous expression of the Ku70 and Ku80 proteins in *E. coli*. Proteins were chromatographically purified with a 6His-tag at the N terminus of the Ku80 subunit. Recombinant Ku protein was produced in bacterial cell culture, purified, and its DNA-binding activity was characterized [28].

Isolation was performed using affinity chromatography on Ni^2+^-NTA resin. The presence of Ku and impurities was monitored using SDS-PAGE and Western blot analysis. The testing of substances as potential inhibitors of the interaction of the Ku heterodimer with DNA was carried out using the gel inhibition assay [29].

The testing of substances as potential inhibitors of the interaction of the Ku heterodimer with DNA was carried out using the EMSA. A 12.8 nM heterodimer Ku, 4 nM radioactively labelled 40 mer DNA duplex, containing 5′-GACTACGGTTCAAGTCAGCGTGTGGAAAATCTCTAGCAGT-3′ and 5′-CTGATGCCAAGTTCAGTCGCACACCTTTTAGAGATCGTCA-3′, were incubated for 20 min at 25 °C with different concentration of inhibitors in 25 mM Tris-HCl pH 8.0, 90 mM KCl, 0.125 mM DTT, 2.5% glycerol, and 10 mM EDTA and then applied to 5% native PAGE. The result was visualized by radioautography on a Typhoon FLA 9500 PhosphorImager (GE Healthcare Technologies, Chicago, IL, USA). The counting of radioautograms was performed using QuantityOne 4.6.6 software (Bio-Rad, Hercules, CA, USA). The calculation of IC_50_ parameters was carried out by Prism 8.0.1 software (GraphPad Software, San-Diego, CA, USA).

### 3.5. Screening Inhibition of HIV Rev–RNA Complex

The rev-coding sequence was amplified with a (His6)-tag at the C-terminus of the protein from the commercial plasmid pRSV-Rev (#12253, Addgene, Watertown, MA USA). To obtain the target plasmid pET-21d-2C-rev, the amplified fragment of the rev gene was cloned into the pET-21d-2C vector [30] at NdeI and XhoI sites. To obtain an RNA containing the RRE, we created a plasmid that allows us to obtain such a transcript. The recombinant *Rev* protein was produced in *E. coli* cell culture, purified, and its DNA-binding activity was characterized [19]. The fragment carrying the RRE element was obtained from the commercial plasmid pRSV-Rev (Addgene, Watertown, MA, USA). The resulting fragment was cloned into the commercial linearized vector pAL2-T (Evrogen, Moscow, Russia) to form the transcription vector pAL2-T-RRE. An RRE-containing virus RNA fragment was synthesized by run-off transcription in vitro using T7 RNA polymerase (TranscriptAid T7 High Yield Transcription Kit, Thermo Fischer Scientific, Waltham, MA, USA) from linearized vector pAL2-T-RRE. Isolation, [α-^32^P]-labeling was performed according to the published procedure [19]. RNA binding Rev activity was evaluated using electrophoretic mobility shift assay, compound testing was performed according to the previously described method [19]. Radiolabeled RNA was heated to 70 °C and snap-cooled on ice. Afterwards, the radiolabeled-RNA was adjusted to the final concentration of 2 nM in 1X RBB buffer (10 mM HEPES, pH 7.6, 150 mM KCl, 5 mM DTT, 10% glycerol, 100 μg/mL BSA, 1X PIC, 1 unit/μL Ribolock (Thermo Fischer Scientific, Waltham, MA, USA), 100 μg/mL yeast tRNA, 1% Triton X-100). Stocks of Rev protein were diluted also with 1X RBB buffer. Binding reactions were performed by mixing RNA and the protein at the specified concentrations. Pyronin Y and DB_3_(7) (*N*^1^,*N*^9^-bis((6-(4-methylpiperazin-1-yl)-1*H*,3′*H*,3″*H*-[2,5′:2′,5″-terbenzo[*d*]imidazol]-2″-yl)methyl)nonanediamide) were used as a positive control [31]. Compounds stock solutions were diluted in DMSO (BioReagent for molecular biology grade, Sigma-Aldrich, Burlington, MA, USA) in case of **I**–**VI** and Pyronin Y (Sigma-Aldrich, Burlington, MA, USA) and in 10 mM aq. HCl (36.5%, Honeywell, Charlotte, NC, USA) solution in case of DB_3_(7). The compounds were prepared as a 20× stock solution. The final DMSO concentration was 5% *v*/*v*. Then, a Rev protein was added to reach a final concentration of 800 nM. The mix was incubated for 1 h on ice and then applied to 5% native PAGE. The result was visualized by radioautography on a Typhoon FLA 9500 PhosphorImager (GE Healthcare Technologies, Chicago, IL, USA). The counting of the radioautograms was performed using QuantityOne 4.6.6 software (Bio-Rad, Hercules, CA, USA). The calculation of the IC_50_ parameters was carried out by Prism 8.0.1 software (GraphPad Software, San-Diego, CA, USA).

### 3.6. Evaluation of the Compounds Activity Against Human Immunodeficiency Virus (HIV-1) Infected Cells

The human MT-4 lymphoblastoid cells were cultured in RPMI 1640 medium supplemented with 10% fetal bovine serum (FBS) (HyClone, Logan, UT, USA), 2 mM l-glutamine, 50 U/mL penicillin, and 50 μg/mL streptomycin in a humidified atmosphere of 5% CO_2_ at 37 °C. The cells were re-seeded at a density of 3.0 × 10^5^ cells/mL every three days. The HIV-1_899A_ strain (subtype B) was used from the collection of human immunodeficiency virus strains of the D.I. Ivanovsky Institute of Virology, N. F. Gamaleya National Research Center of Epidemiology and Microbiology, Ministry of Health, Moscow, Russia. The commercial drug Efavirenz (EFV), a non-nucleoside inhibitor of HIV reverse transcriptase, was used as a positive control. HIV-1 was added to the cells at 0.1 CCID_50_ (50% cell culture infectious dose). The cells, both infected and non-infected, were then incubated at 37 °C in a humidified atmosphere containing 5% CO_2_ for a 40 min period, after which the cells were washed twice in serum-free RPMI 1640 and plated into the wells of a 96-well plate. The tested compounds were then added to the cells at concentrations from 0.5 μM to 100 μM. The final ethanol concentration was 1% *v*/*v*. On the third day after infection, the culture fluid was collected for determining the amount of p24 antigen by enzyme immunoassay analysis using a commercial test system kit (Genscreen ULTRA HIV Ag-Ab, Bio-Rad, Hercules, CA, USA). Cell viability was measured by MTT assay [32].

### 3.7. Bacterial and Fungi Culture

*Candida albicans* ATCC 10231, *Pseudomonas aeruginosa* ATCC 27853, and *Staphylococcus aureus* ATCC 29213 strains were obtained from the American Type Culture Collection (ATCC; Rockville, MD, USA). The subculture and inoculum preparation were performed according to CLSI M27-A3 for *C. albicans* on yeast extract peptone dextrose (YPD) agar (HiMedia, Mumbai, Maharashtra, India) and RPMI-1640 medium (PanEco, Moscow, Russia) with MOPS (3-(*N*-morpholino) propanesulfonic acid, >99.5%; PanEco, Moscow, Russia) [33], and M7-A11 for *P. aeruginosa* and *S. aureus* on Mueller-Hinton agar (MHA; HiMedia, Mumbai, Maharashtra, India) and Mueller-Hinton broth (MHB; HiMedia, Mumbai, Maharashtra, India) [34]. The stock cell concentrations of fungus and bacteria were 1 × 10^6^ and 1 × 10^8^ cells/mL, respectively. A working suspension of *C. albicans* (5.0 × 10^2^ cells/mL) was prepared by two-step dilution of the broth concentration with the RPMI-1640 medium. Assay suspensions of *P. aeruginosa* and *S. aureus* (1.0 × 10^6^ cells/mL) were prepared by diluting the concentration with Mueller-Hinton broth (HiMedia, Mumbai, Maharashtra, India). Amphotericin B (Sigma-Aldrich, Burlington, MA, USA) and gentamicin (Sigma-Aldrich, Burlington, MA, USA) were used as secondary pharmaceutical standards.

### 3.8. Antimicrobial Activity Evaluation

The minimum inhibitory concentration (MIC) was determined by the twofold serial dilution method, according to CLSI M27-A3 [33] and M07-A11 [34], in three independent experiments with four replicates each. Amphotericin B was used as a positive control for fungus; gentamicin was used as a positive control for bacteria. Stock solutions except gentamicin were prepared in DMSO at a concentration of 6400 μg/mL. Gentanicin was solubilized in sterile water at a concentration of 1280 μg/mL. Stock solutions of the tested substances were diluted to the final concentrations in the assay medium and 100 μL of each sample was added to the wells of 96-well plates, and then 100 μL of inoculum was added. Rows 11 and 12 were left for the negative control (medium with inoculum without the drug) and sterility control. The plates were incubated for 24 h at 35 °C in the case of *C. albicans* and at 37 °C in the case of *P. aeruginosa* and *S. aureus*. The tested concentration for gentamicin ranged from 0.0625 to 32 μg/mL; for other compounds, it ranged from 0.125 to 64 μg/mL. The MIC value was determined using spectrophotometer. The MIC was taken to be the lowest drug concentration that caused complete inhibition of microorganism growth compared to the control. The final MIC values were reported as the means with standard error (SE). Standard errors for µg/mL values less than 0.01 were excluded; standard errors for µM values less than 0.1 were excluded (Table 3).

### 3.9. Microgel Formation

Commercially available alginate extracted from the brown algae *Laminaria* species was used, filtered, and a CuSO_4_·5H_2_O (Reakhim, Moscow, Russia) solution was added to the precipitate the alginate. The alginate salt was collected and converted into alginic acid by treating the polysaccharide with dilute HCl (5% *w*/*v*). After purification from doubly charged ions impurities, a water-soluble powder of sodium alginate was obtained.

The expected mass loading of microgels was 33% (5.0 mg of alginate + 2.5 mg of a sample). A solution of sodium alginate was prepared at a concentration of 3.0 mg/mL. 1.700 mL of sodium alginate solution and 3.283 mL of distilled water were added to the beaker and an equimolar copper sulfate solution (17.0 μL) was added dropwise and stirred for 30 min. Then, concentrated hydrochloric acid was dissolved in 250 μL of the polymer solution for 20 min while heating to 50 ˚C until the solid residue was completely dissolved. The resulting solution of **I** was added dropwise to the Cu^2+^–alginate microgel solution and stirred for 24 h; then, the acid was neutralized with a concentrated NaOH aq. solution. The resulting solutions were purified using flow dialysis. Microgels with substances were lyophilized until a fully dry pinkish powder was obtained.

To confirm the invariability of substances in the course of creating microgel adsorbed forms, we simulated the conditions of the adsorption procedure without the addition of Cu^2+^–alginate microgels. In the general case, the substances were subjected to the same effects, which made it possible to study their stability according to NMR spectroscopy data. A 0.8 mg portion of one of the samples **I** was dissolved in 550 μL of DMSO-*d*_6_, and ^1^H NMR spectrum was recorded. In parallel, the substance **I** was treated with a solution of concentrated hydrochloric acid at 50 °C for 20 min until complete dissolution, then neutralized to pH 7; the remaining sodium chloride was removed by filtration. The resulting precipitate was dissolved in DMSO-*d*_6_ and the NMR spectrum was recorded and analyzed for consistency with the starting hydrazone.

### 3.10. Cell Culture for Cytotoxicity Assays

Huh7.5 cells were grown in the DMEM/F12 (2:1) medium supplemented with 10% FBS (BioSera, Cholet, France), 2 mM l-glutamine, 100 U/mL penicillin, and 100 µg/mL streptomycin in the presence of 5% CO_2_ at 37 °C. Cells were re-seeded every three days at a ratio of 1:3 or 1:5.

### 3.11. MTT Assay

Stock solutions were diluted in DMSO (BioReagent of molecular biology grade, Sigma, St. Louis, MO, USA), and the final DMSO concentration was 1%. After 24 h (30–40% monolayer), the solutions of the studied compounds diluted at different concentrations were introduced to the culture medium. After incubation at 37 °C in 5% CO_2_ for 48 h, a freshly prepared solution of thiazolyl blue tetrazolium bromide at a final concentration of 0.4 mg/mL was added to each well of the plate. Incubation was performed at +37 °C for 2 h. The medium containing unreacted MTT was removed via aspiration. The formed formazan dye was dissolved in 100 µL of isopropanol added to each well of the plate. The result of the analysis was evaluated using a Tecan Spark multimode microplate reader (Tecan Group Ltd., Männedorf, Switzerland) via absorbance at 544 nm.

### 3.12. Neutral Red (NR) Assay

The 40 µg/mL neutral red working solution was prepared, incubated overnight at the same temperature as the cells, and then centrifuged for 10 min at 600× *g* to remove any precipitated dye crystals. After the incubation with compounds tested at 37 °C in 5% CO_2_ for 48 h, cells were washed with PBS and then 100 µL of neutral red medium was added to each well of the plate. The plate was incubated for 2 h at the appropriate culture conditions, then the neutral red medium was removed and the cells were washed with PBS. After that 150 µL neutral red destain solution per well was added (50% ethanol, 49% deionized water, 1% glacial acetic acid (Sigma-Aldrich, Burlington, MA, USA). After the neutral red was extracted from the cells and formed a homogeneous solution, the result of the analysis was evaluated using a Tecan Spark multimode microplate reader via absorbance at 540 nm [35].

### 3.13. Sulforhodamine B (SRB) Assay

After an incubation period, cell monolayers were fixed with 10% (*w*/*v*) trichloroacetic acid and stained for 30 min by adding 100 µL of 0.057% (*w*/*v*) SRB solution to each well, after which the excess dye is removed by washing repeatedly with 1% (*v*/*v*) acetic acid. The protein-bound dye is dissolved in 10 mM Tris-base solution with pH 10.5 for OD determination using a Tecan Spark multimode microplate reader via absorbance at 510 nm [36].

### 3.14. Statistical Analysis

All experiments were performed in triplicate. The results were reported as the mean ± standard error (SE) using the MS Excel Statistical Analysis tool pack.

## 4. Conclusions

To summarize, we have assessed the antipathogenic potential of a series of aromatic hydrazones based on 7-hydrazino-8-hydroxyquinoline. The compounds demonstrate biological activity against both viral and microbial targets, showing their versatility as potential antimicrobial and antiviral agents. Notably, this class was found to possess Ku70-inhibiting activity with promising implications for anti-HIV therapeutic development. HIV p24-assay demonstrated activity at sub-micromolar concentrations, confirming that the compounds act as multitarget antiviral agents. Among the tested microbial pathogens, hydrazones of 7-hydrazo-8-hydroxyquinoline possessed significant activity at low concentrations against fungus *C. albicans*. All these findings may lead to more focused modifications in the search for novel multitarget pharmacophores. We have tried to resolve the water solubility issue, which is a serious obstacle in biological testing. The solubility of the hydrazones was successfully enhanced through their incorporation into a water-soluble polyalginate microgel, revealing the potential of these compounds for practical applications. These findings can serve as a basis for further optimization of 7-hydrazo-8-hydroxyquinoline derivatives and investigation of their mechanism of action.

## Figures and Tables

**Table 1:** Compounds inhibition of HIV integrase strand transfer.

Compound	I	II	III	IV	V	VI
IC_50_, μM	160 ± 50	>200	190 ± 60	150 ± 50	>200	40 ± 10

**Table 2:** Compounds inhibition of of Ku70–DNA complex formation.

Compound	I	II	III	IV	V	VI
IC_50_, μM	40 ± 10	50 ± 20	60 ± 20	80 ± 30	25 ± 8	>250

**Table 3:** MICs values of the hydrazones against *C. albicans*, *P. aeruginosa,* and *S. aureus*.

Compounds	MIC (Mean ± SE) *					
	***C. albicans*** ATCC 10231		***S. aureus*** ATCC 29213		***P. aeruginosa*** ATCC 27853	
	μg/mL	μM	μg/mL	μM	μg/mL	μM
**I**	4.0	15.2	52.0 ± 5.9	197.5 ± 22.3	>64	>243
**II**	4.0	13.0	14.0 ± 1.9	45.4 ± 6.2	>64	>208
**III**	11.3 ± 1.2	36.8 ± 3.9	18.0 ± 4.8	58.4 ± 15.6	>64	>208
**IV**	8.0	27.3	64.0	218.2	>64	>218
**V**	15.3 ± 0.7	36.3 ± 1.7	36.3 ± 10.3	86.1 ± 24.5	>64	>152
**VI**	3.8 ± 0.2	13.6 ± 0.6	≥64	≥227.5	>64	>228
Amphotericin B	0.4	0.4	n/d	n/d	n/d	n/d
Gentamicin	n/d **	n/d	0.6 ± 0.1	1.3 ± 0.1	2.0 ± 0.3	4.2 ± 0.6

* The error value is not included in cases where it is less than 0.05; ** n/d—not determined.

**Table 4:** CC_50_ of compounds in different cell proliferation assays in Huh-7.5 cells.

Compound	MTT	NR	SRB
**I**	50.5 ± 2.5	14.5 ± 3.5	59.5 ± 12.5
**II**	123.0 ± 13.2	21.0 ± 4.0	8.0 ± 0.1
**III**	38.0 ± 9.1	7.1 ± 0.7	7.1 ± 0.4
**IV**	47.0 ± 3.2	9.1 ± 1.5	40.0 ± 0.2
**V**	120.5 ± 7.5	8.6 ± 0.1	6.9 ± 0.3
**VI**	45.5 ± 3.6	6.7 ± 0.2	7.0 ± 0.2
**I** in alginate microgel	>150	>150	>150

## Data Availability

The original contributions presented in this study are included in the article/Supplementary Materials. Further inquiries can be directed to the corresponding author.

## Supplementary Materials

The following supporting information can be downloaded at: https://www.mdpi.com/article/10.3390/ijms26178402/s1.
