# Supplementary material for: Anti-HIV and Antimicrobial Activity of 7-Hydrazino-8-hydroxyquinoline-Based Aromatic Hydrazones
Source: Int J Mol Sci. 2025 Aug 29;26(17):8402. doi: 10.3390/ijms26178402 (PMC12428554; doi:10.3390/ijms26178402)
Supplement: Supplementary file 1 [file ijms-26-08402-s001.zip › ijms-3716260-supplementary.pdf]

ELECTRONIC SUPPLEMENTARY MATERIAL

Anti-HIV and Antimicrobial Activity of 7-Hydrazino-8-hydroxyquinoline-Based Aromatic Hydrazones

Yaroslav V. Kozmenko,[1,] Marat M. Khisamov [1], Svetlana V. Revtovich [1], Sergey P. Korolev [2,3], Daria K. Sherman [1], Vasiliy V. Spiridonov [2,3], Lyudmila B. Kalnina [4], Vladimir T. Valuev-Elliston [1], Marina B. Gottikh [2,3], Sergey N. Kochetkov [1,2], Anastasia S. Zemskaya [1] and Pavel N. Solyev [1,*]

[1] Engelhardt Institute of Molecular Biology, Moscow 119991, Russia; yrko2002@gmail.com (Y.V.K.); hisamovmaratag@mail.ru (M.M.K.); svetla21@mail.ru (S.V.R.); dar.sher.man0@gmail.com (D.K.S.); gansfaust@mail.ru (V.T.V.-E.); snk1952@gmail.com (S.N.K.); a.zemskaia@mail.ru (A.S.Z.)
[2] Chemistry Department, Lomonosov Moscow State University, Moscow 119992, Russia; spkorolev@mail.ru (S.P.K.); vasya_spiridonov@mail.ru (V.V.S.)
[3] Belozersky Research Institute of Physico-Chemical Biology, Lomonosov Moscow State University, Moscow 119992, Russia; gottikh@belozersky.msu.ru
[4] Ivanovsky Institute of Virology, N. F. Gamaleya National Research Center of Epidemiology and Microbiology, Moscow 123098, Russia; klb3@yandex.ru

* Correspondence: solyev@gmail.com

Figures S1-S6. HPLC-HRMS analysis of the compounds **I** – **VI**-------------------------------- 2-7
Figure S7. ^{1}H NMR spectrum of the compound **I** (*anti*- + ~20% *syn*-) -------------------------- 8
Figure S8. ^{13}C NMR spectrum of the compound **I** (*anti*- + ~20% *syn*-) ------------------------- 9
Figure S9. ^{1}H-^{13}C HSQC NMR spectrum of the compound **I** (*anti*- + ~20% *syn*-) ------------ 10
Figure S10. ^{1}H NMR spectrum of the compound **II** (*anti*- + ~40% *syn*-) ----------------------- 11
Figure S11. ^{13}C NMR spectrum of the compound **II** (*anti*- + ~40% *syn*-)----------------------- 12
Figure S12. ^{1}H-^{13}C HSQC NMR spectrum of the compound **II** (*anti*- + ~20% *syn*-) --------- 13
Figure S13. ^{1}H NMR spectrum of the compound **III** (*anti*- + ~10% *syn*-) --------------------- 14
Figure S14. ^{13}C NMR spectrum of the compound **III** (*anti*- + ~10% *syn*-) -------------------- 15
Figure S15. ^{1}H-^{13}C HSQC NMR spectrum of the compound **III** (*anti*- + ~10% *syn*-) -------- 16
Figure S16. ^{1}H NMR spectrum of the compound **IV** (*anti*- + ~10% *syn*-) --------------------- 17
Figure S17. ^{13}C NMR spectrum of the compound **IV** (*anti*- + ~10% *syn*-) -------------------- 18
Figure S18. ^{1}H-^{13}C HSQC NMR spectrum of the compound **IV** (*anti*- + ~10% *syn*-) -------- 19
Figure S19. ^{1}H NMR spectrum of the compound **V** (*anti*- + ~10% *syn*-) ----------------------- 20
Figure S20. ^{13}C NMR spectrum of the compound **V** (*anti*- + ~10% *syn*-)----------------------- 21
Figure S21. ^{1}H-^{13}C HSQC NMR spectrum of the compound **V** (*anti*- + ~10% *syn*-)---------- 22
Figure S22. ^{1}H NMR spectrum of the compound **VI** (*anti*- + ~15% *syn*-) --------------------- 23
Figure S23. ^{13}C NMR spectrum of the compound **V** (*anti*- + ~15% *syn*-)----------------------- 24
Figure S24. ^{1}H-^{13}C HSQC NMR spectrum of the compound **V** (*anti*- + ~15% *syn*-)---------- 25
Figure S25. EMSA analysis of Ku/DNA interaction inhibition by **V** --------------------------- 26
Figure S26. EMSA analysis of Rev–RNA complex interaction with hydrazones **I-VI**-------- 26
Figure S27. Effects of compound **VI** and EFV on syncytia formation--------------------------- 26
Figure S28. UV-Vis absorption spectra of 7-hydrazo-8-hydroxyquinoline hydrazones ------- 27
Figure S29. 7-Hydrazo-8-hydroxyquinoline hydrazones in diluted methanol solutions ------ 28
Figure S30. Calibration curves of the compounds at selected λ_{max} ------------------------------- 25
Table S1. Calculated data for hydrazone incorporated microgel formation -------------------- 28
Figure S31. ^{1}H-NMR spectrum of the hydrazone **II** (a mixture of tautomeric forms in DMSO-d_6) (A) and the sample of **II** incorporated into the alginate microgel (B)--------------------------- 28
Figure S32. IR spectrum of alginate microgel without included substance (1), hydrazone **II** (2), and alginate microgel with incorporated hydrazone **II** (3)-- 28

Figure S1. HPLC-HRMS analysis of the compound **I**.

Display Report

Analysis Info

Analysis Name	D:\Data\PS-286 (hydr-I)_61_01_5666.d	Acquisition Date	8/8/2025 7:24:04 PM
Method	la_2.2_small2-nonfrg.m	Operator	BDAL@DE
Sample Name	PS-286 (hydr-I)	Instrument	compact 8255754.20088
Comment			

Acquisition Parameter

Source Type	ESI	Ion Polarity	Positive	Set Nebulizer	0.4 Bar
Focus	Active	Set Capillary	4300 V	Set Dry Heater	180 °C
Scan Begin	50 m/z	Set End Plate Offset	-500 V	Set Dry Gas	4.0 l/min
Scan End	3000 m/z	Set Charging Voltage	2000 V	Set Divert Valve	Source
		Set Corona	0 nA	Set APCI Heater	0 °C

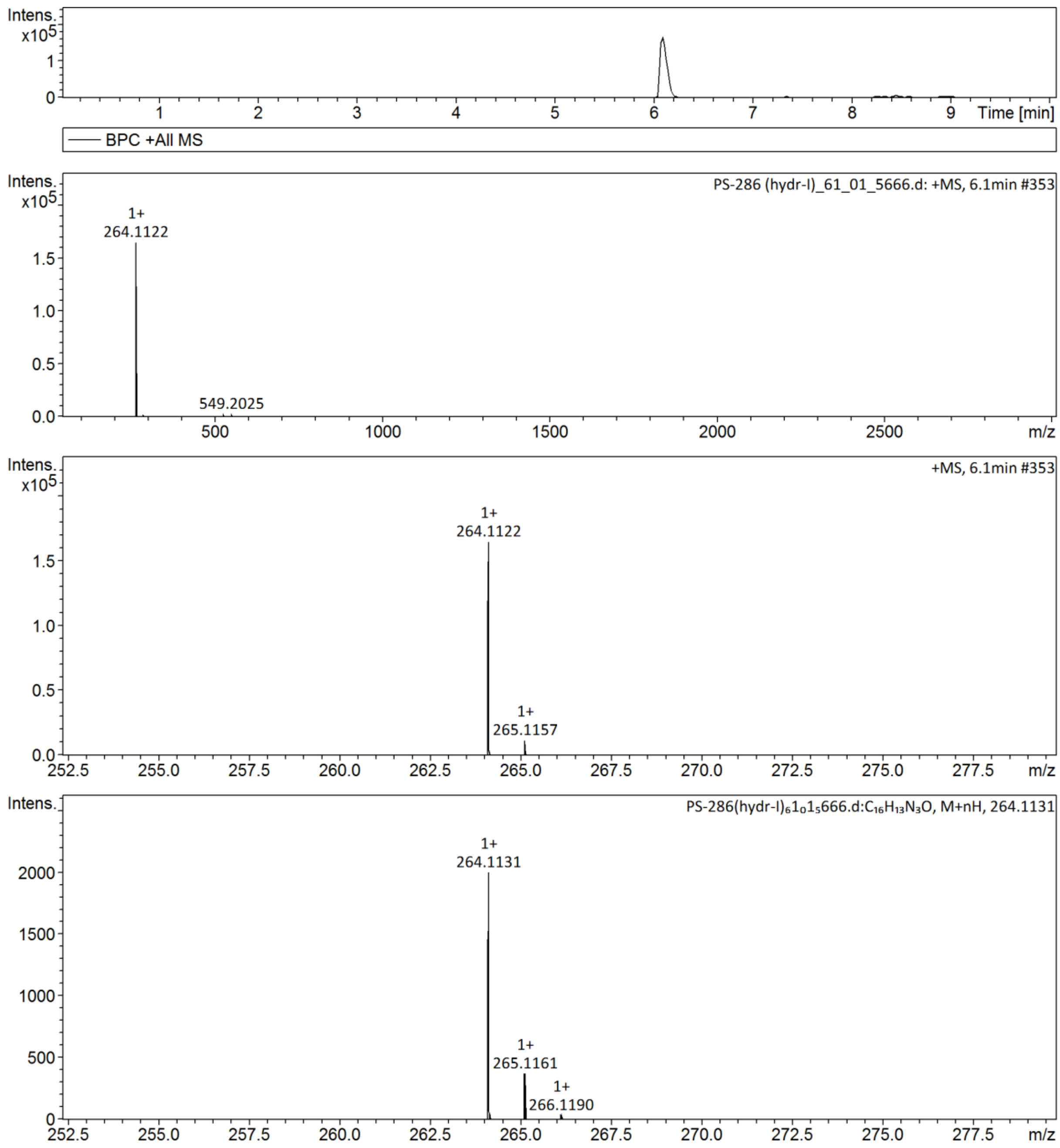

PS-286 (hydr-I)_61_01_5666.d

Bruker Compass DataAnalysis 4.3 printed: 8/8/2025 8:53:26 PM by: BDAL@DE Page 1 of 1

Figure S2. HPLC-HRMS analysis of the compound **II**.

Display Report

Analysis Info

Analysis Name	D:\Data\PS-287 (hydr-II)_62_01_5667.d	Acquisition Date	8/8/2025 7:35:36 PM
Method	la_2.2_small2-nonfrg.m	Operator	BDAL@DE
Sample Name	PS-287 (hydr-II)	Instrument	compact 8255754.20088
Comment			

Acquisition Parameter

Source Type	ESI	Ion Polarity	Positive	Set Nebulizer	0.4 Bar
Focus	Active	Set Capillary	4300 V	Set Dry Heater	180 °C
Scan Begin	50 m/z	Set End Plate Offset	-500 V	Set Dry Gas	4.0 l/min
Scan End	3000 m/z	Set Charging Voltage	2000 V	Set Divert Valve	Source
		Set Corona	0 nA	Set APCI Heater	0 °C

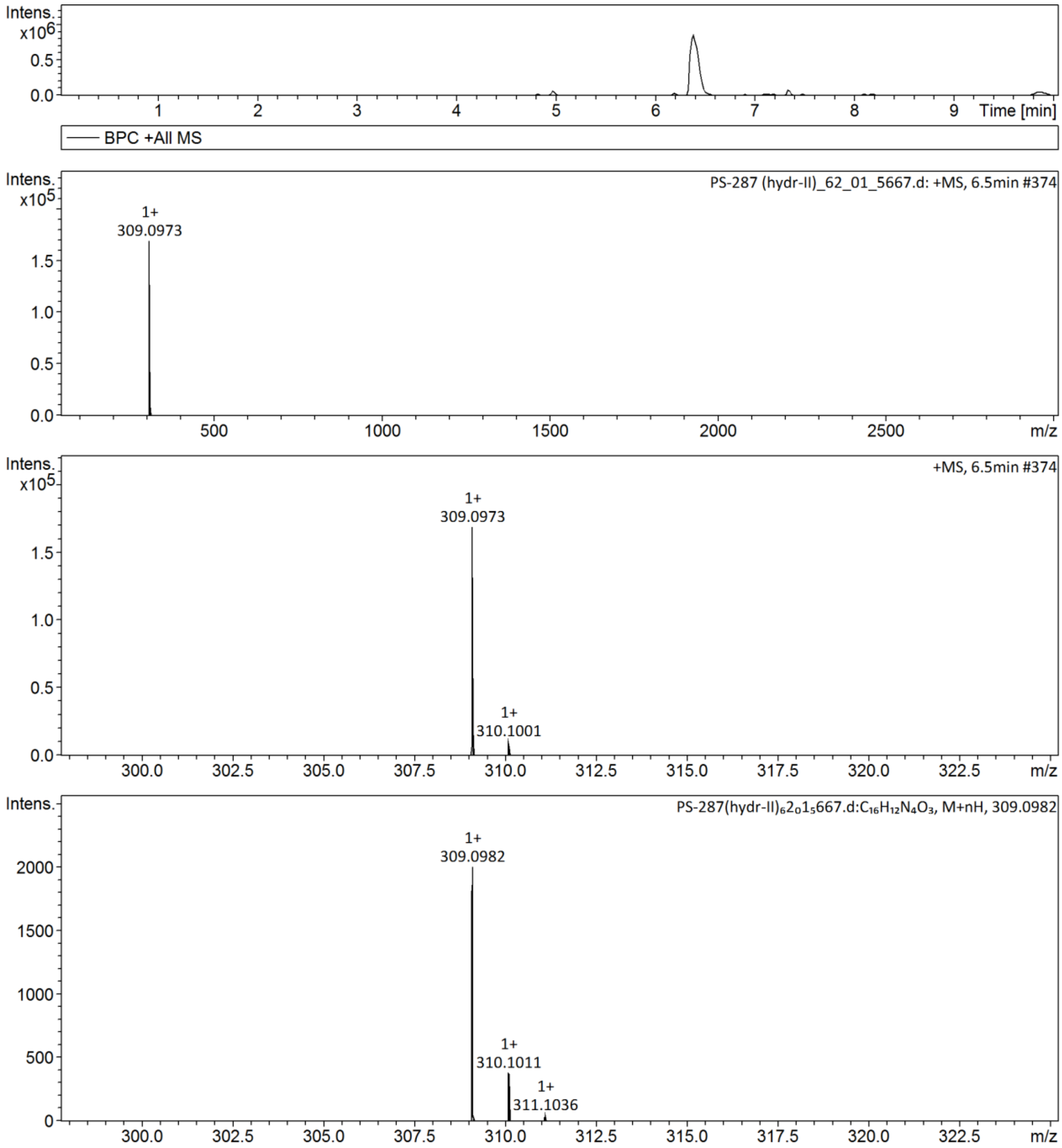

PS-287 (hydr-II)_62_01_5667.d

Bruker Compass DataAnalysis 4.3 printed: 8/8/2025 8:54:52 PM by: BDAL@DE Page 1 of 1

Figure S3. HPLC-HRMS analysis of the compound **III**.

Display Report

Analysis Info

Analysis Name	D:\Data\PS-288 (hydr-III)_63_01_5668.d	Acquisition Date	8/8/2025 7:47:11 PM
Method	la_2.2_small2-nonfrg.m	Operator	BDAL@DE
Sample Name	PS-288 (hydr-III)	Instrument	compact 8255754.20088
Comment			

Acquisition Parameter

Source Type	ESI	Ion Polarity	Positive	Set Nebulizer	0.4 Bar
Focus	Active	Set Capillary	4300 V	Set Dry Heater	180 °C
Scan Begin	50 m/z	Set End Plate Offset	-500 V	Set Dry Gas	4.0 l/min
Scan End	3000 m/z	Set Charging Voltage	2000 V	Set Divert Valve	Source
		Set Corona	0 nA	Set APCI Heater	0 °C

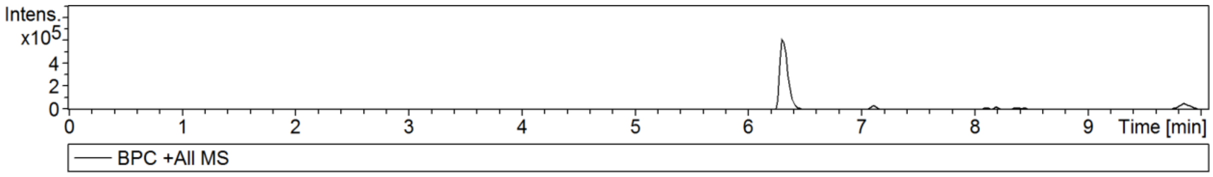

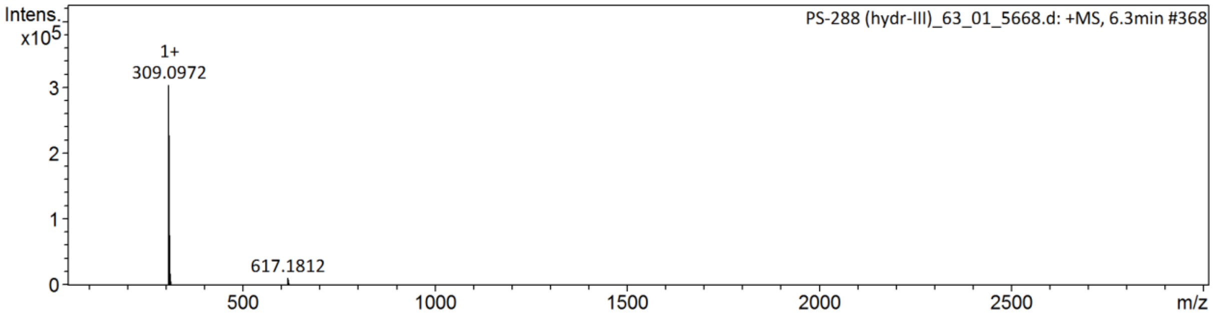

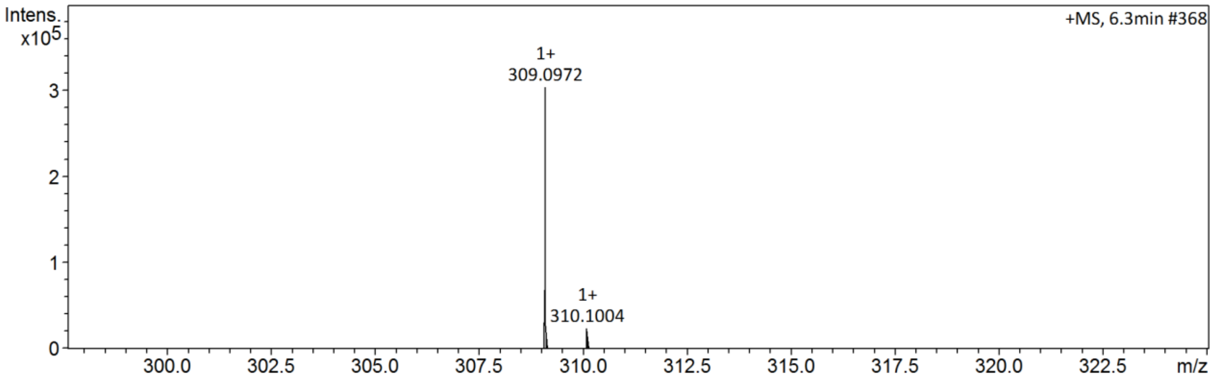

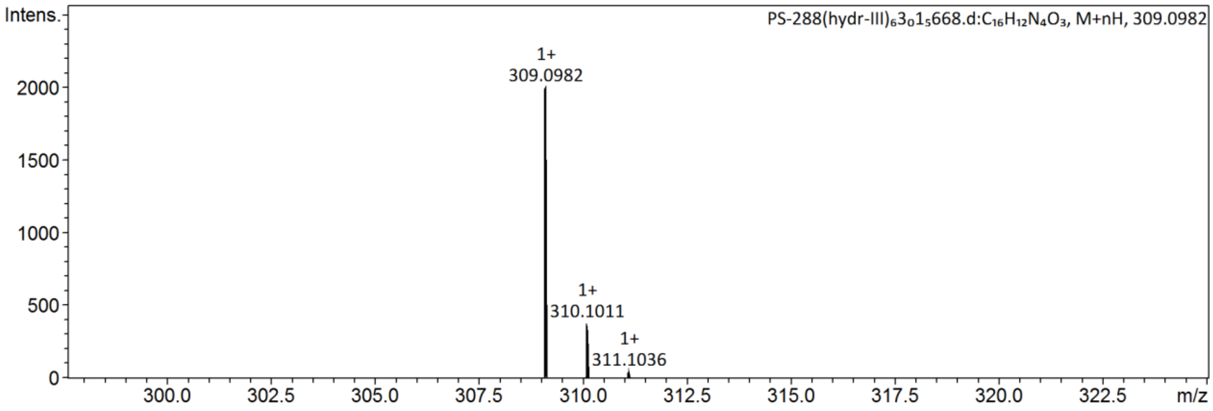

PS-288 (hydr-III)_63_01_5668.d

Bruker Compass DataAnalysis 4.3 printed: 8/8/2025 8:55:50 PM by: BDAL@DE Page 1 of 1

Figure S4. HPLC-HRMS analysis of the compound **IV**.

Display Report

Analysis Info

Analysis Name	D:\Data\PS-289 (hydr-IV)_64_01_5669.d	Acquisition Date	8/8/2025 7:58:43 PM
Method	la_2.2_small2-nonfrg.m	Operator	BDAL@DE
Sample Name	PS-289 (hydr-IV)	Instrument	compact 8255754.20088
Comment			

Acquisition Parameter

Source Type	ESI	Ion Polarity	Positive	Set Nebulizer	0.4 Bar
Focus	Active	Set Capillary	4300 V	Set Dry Heater	180 °C
Scan Begin	50 m/z	Set End Plate Offset	-500 V	Set Dry Gas	4.0 l/min
Scan End	3000 m/z	Set Charging Voltage	2000 V	Set Divert Valve	Source
		Set Corona	0 nA	Set APCI Heater	0 °C

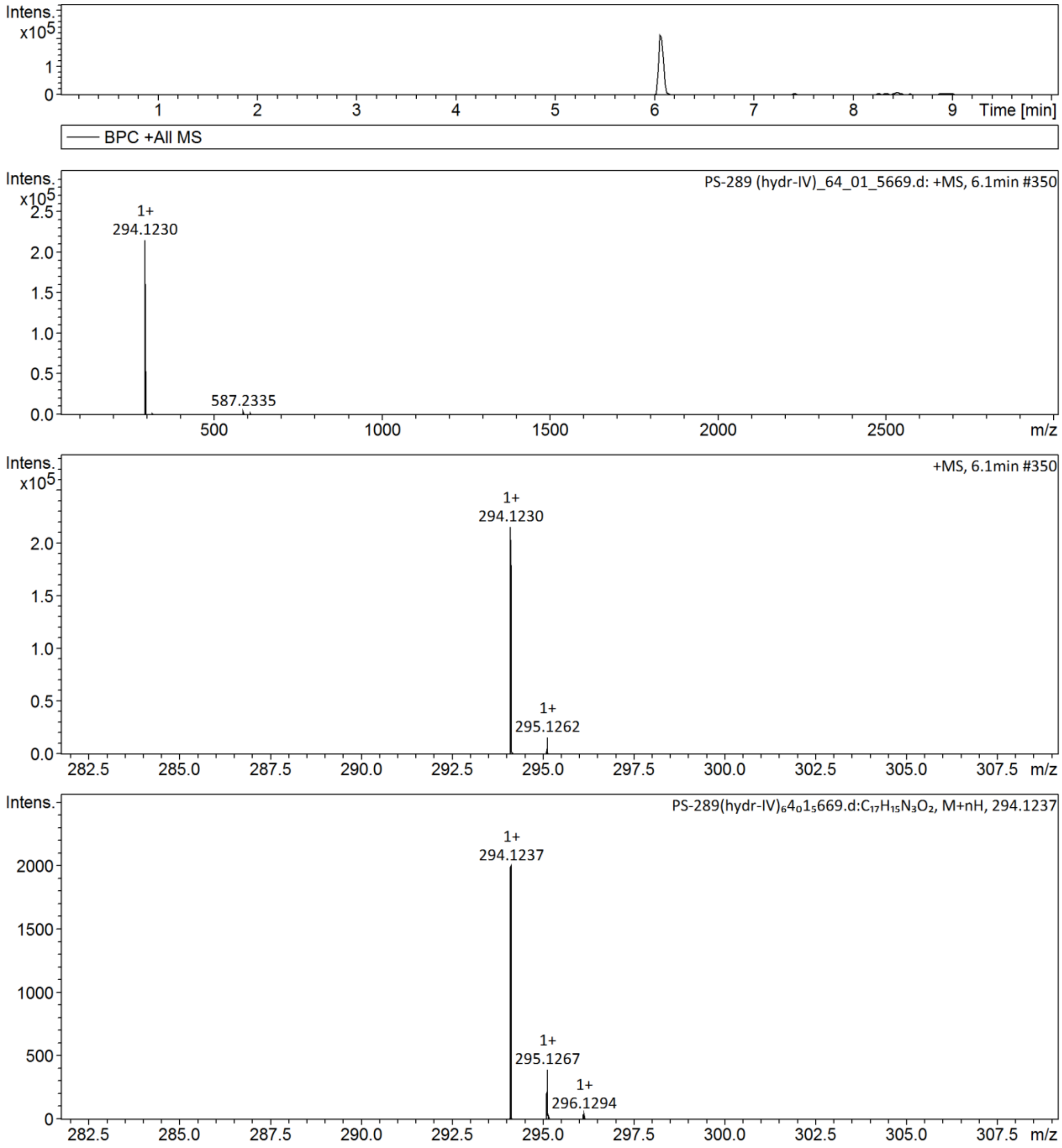

PS-289 (hydr-IV)_64_01_5669.d
Bruker Compass DataAnalysis 4.3 printed: 8/8/2025 8:57:33 PM by: BDAL@DE Page 1 of 1

Figure S5. HPLC-HRMS analysis of the compound **V**.

Display Report

Analysis Info

Analysis Name	D:\Data\PS-290 (hydr-V)_65_01_5670.d	Acquisition Date	8/8/2025 8:10:21 PM
Method	la_2.2_small2-nonfrg.m	Operator	BDAL@DE
Sample Name	PS-290 (hydr-V)	Instrument	compact 8255754.20088
Comment			

Acquisition Parameter

Source Type	ESI	Ion Polarity	Positive	Set Nebulizer	0.4 Bar
Focus	Active	Set Capillary	4300 V	Set Dry Heater	180 °C
Scan Begin	50 m/z	Set End Plate Offset	-500 V	Set Dry Gas	4.0 l/min
Scan End	3000 m/z	Set Charging Voltage	2000 V	Set Divert Valve	Source
		Set Corona	0 nA	Set APCI Heater	0 °C

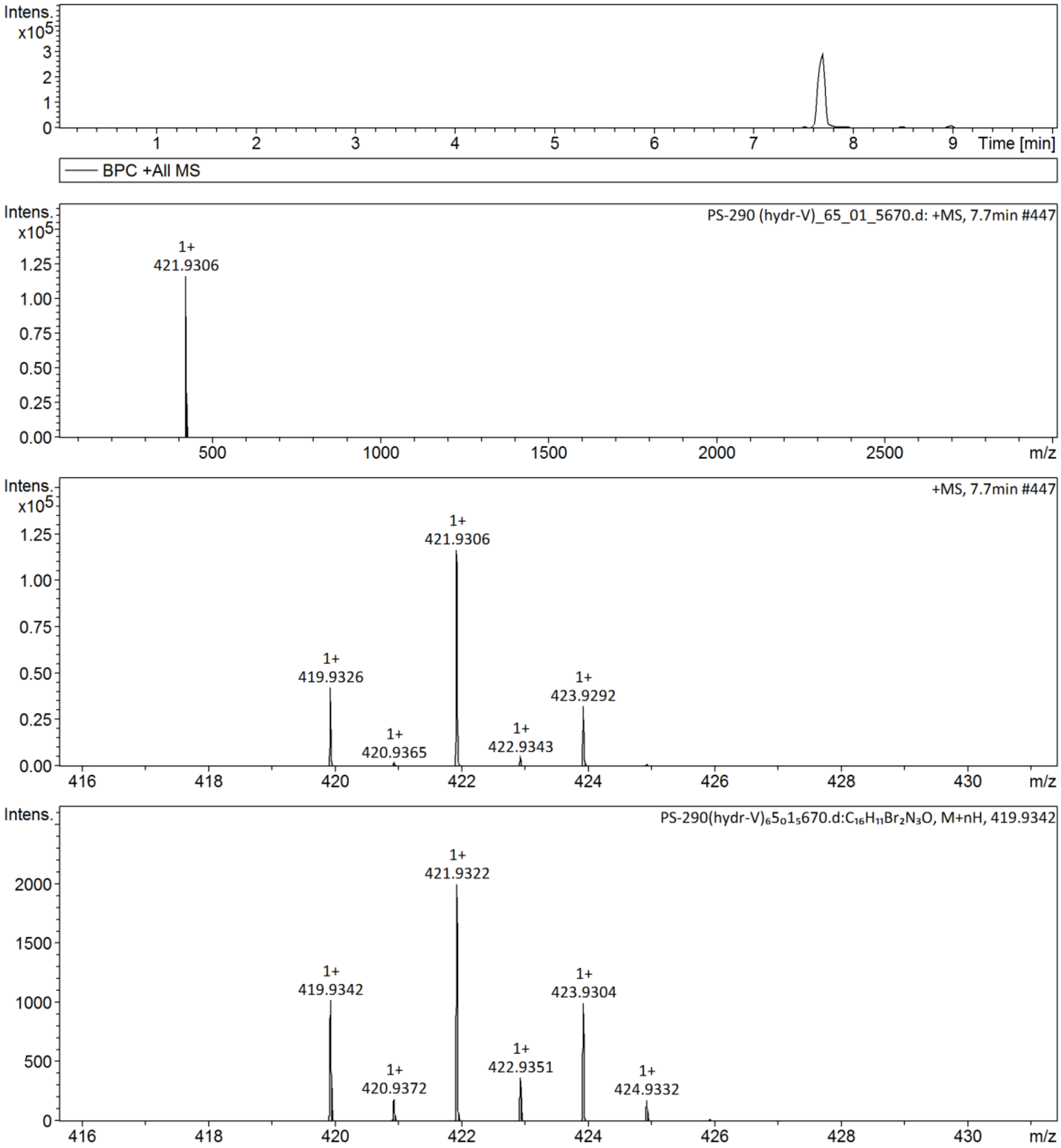

PS-290 (hydr-V)_65_01_5670.d

Bruker Compass DataAnalysis 4.3 printed: 8/8/2025 9:01:46 PM by: BDAL@DE Page 1 of 1

Figure S6. HPLC-HRMS analysis of the compound **VI**.

Display Report

Analysis Info

Analysis Name	D:\Data\PS-291 (hydr-VI)_66_01_5671.d	Acquisition Date	8/8/2025 8:21:56 PM
Method	la_2.2_small2-nonfrg.m	Operator	BDAL@DE
Sample Name	PS-291 (hydr-VI)	Instrument	compact 8255754.20088
Comment			

Acquisition Parameter

Source Type	ESI	Ion Polarity	Positive	Set Nebulizer	0.4 Bar
Focus	Active	Set Capillary	4300 V	Set Dry Heater	180 °C
Scan Begin	50 m/z	Set End Plate Offset	-500 V	Set Dry Gas	4.0 l/min
Scan End	3000 m/z	Set Charging Voltage	2000 V	Set Divert Valve	Source
		Set Corona	0 nA	Set APCI Heater	0 °C

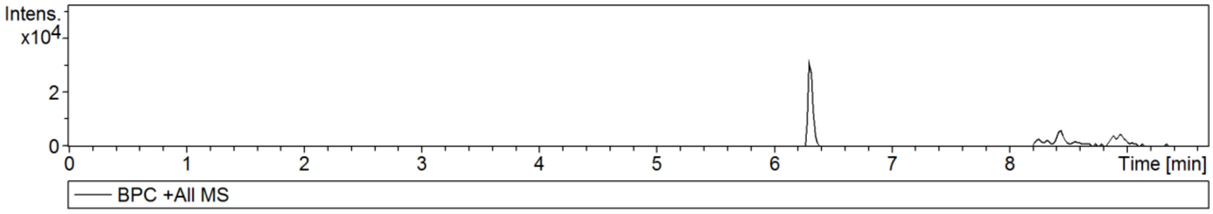

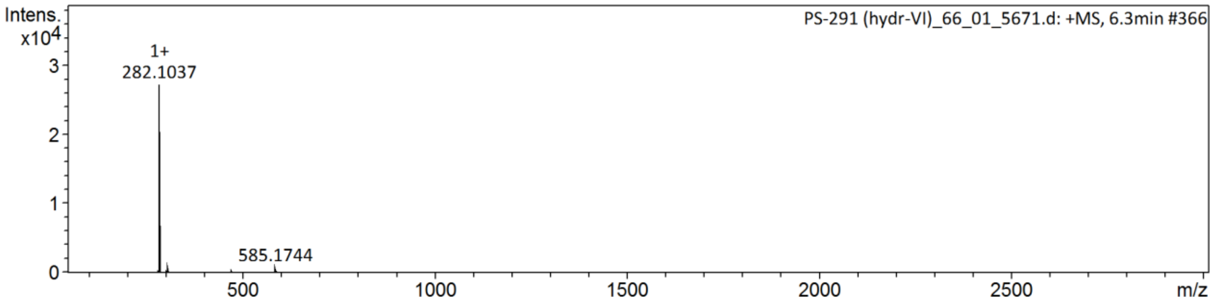

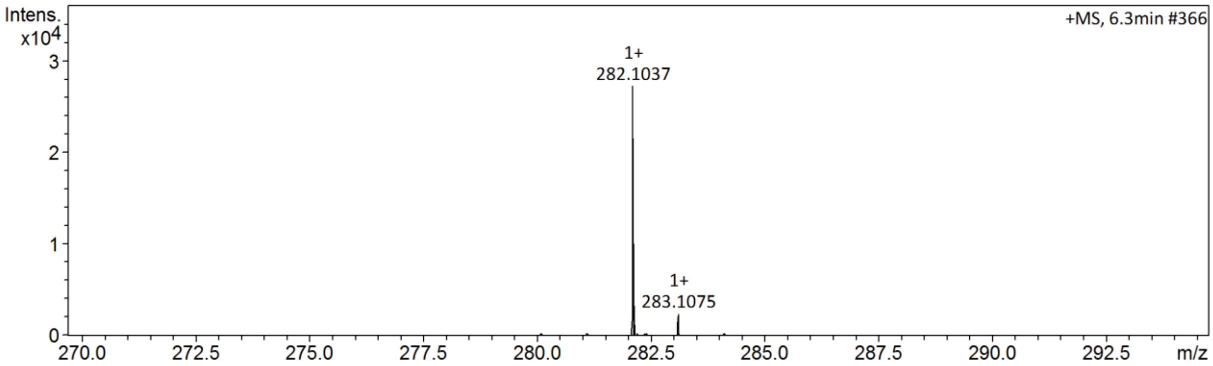

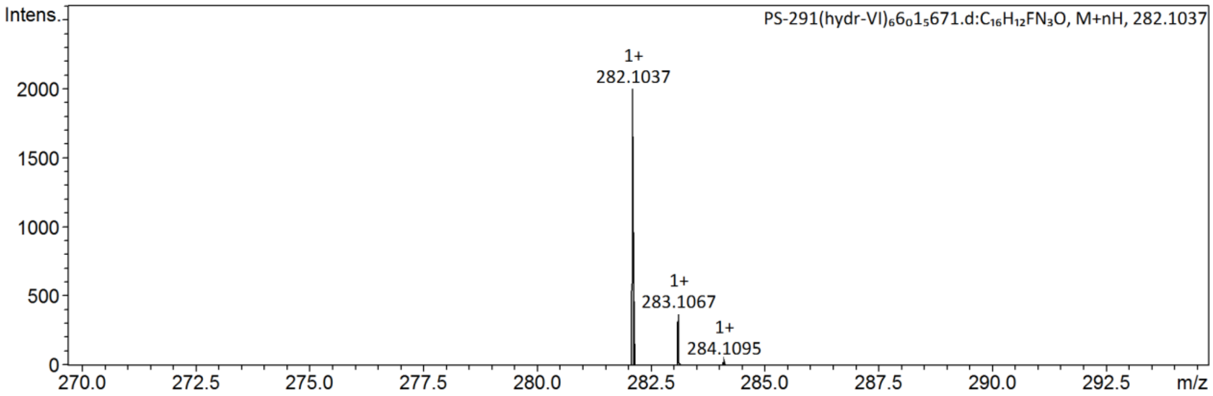

Figure S7. ^{1}H NMR spectrum of the compound **I** (*anti*-isomer + ~20% *syn*-isomer).

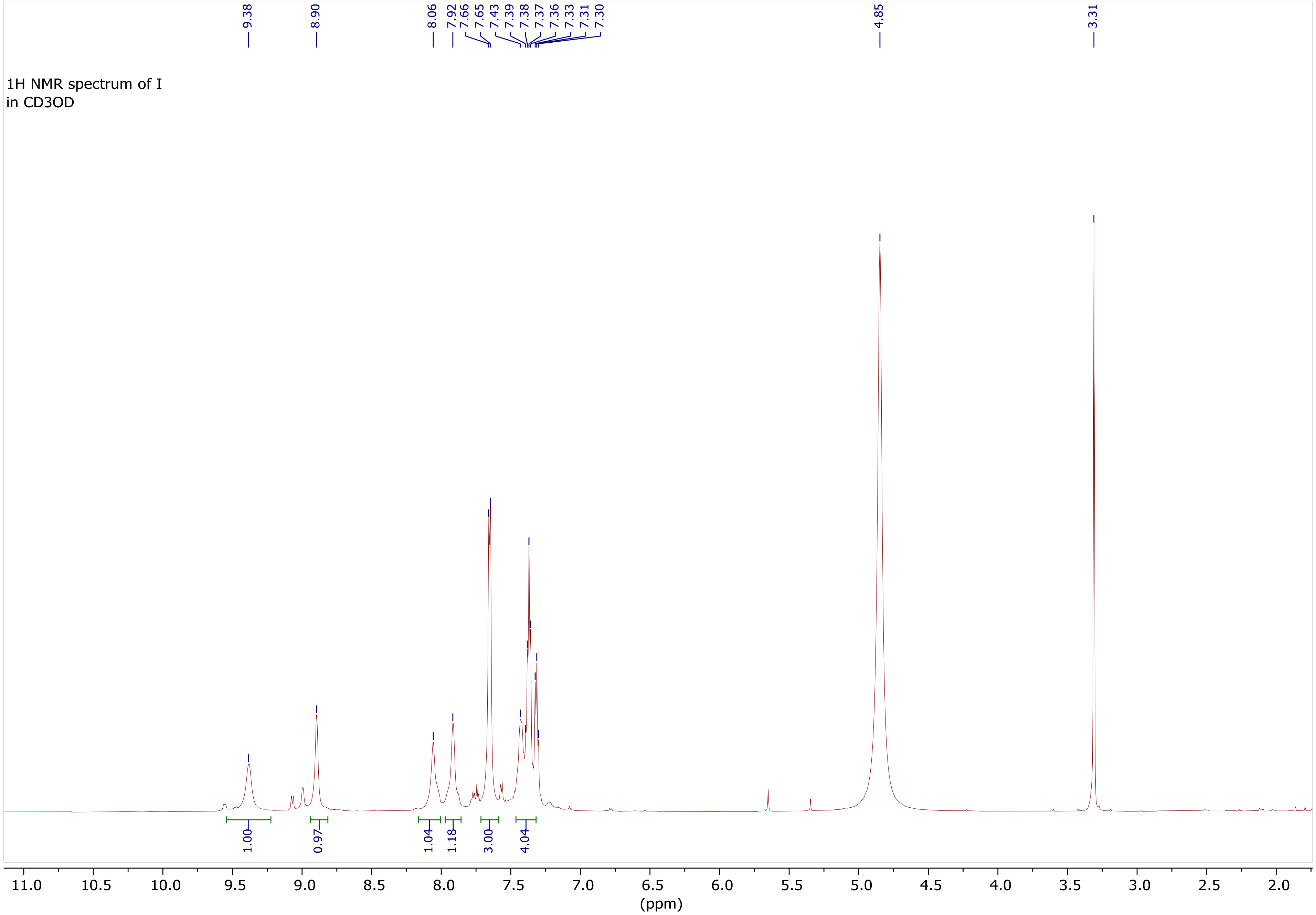

Figure S8. ^{13}C NMR spectrum of the compound **I** (*anti*-isomer + ~20% *syn*-isomer).

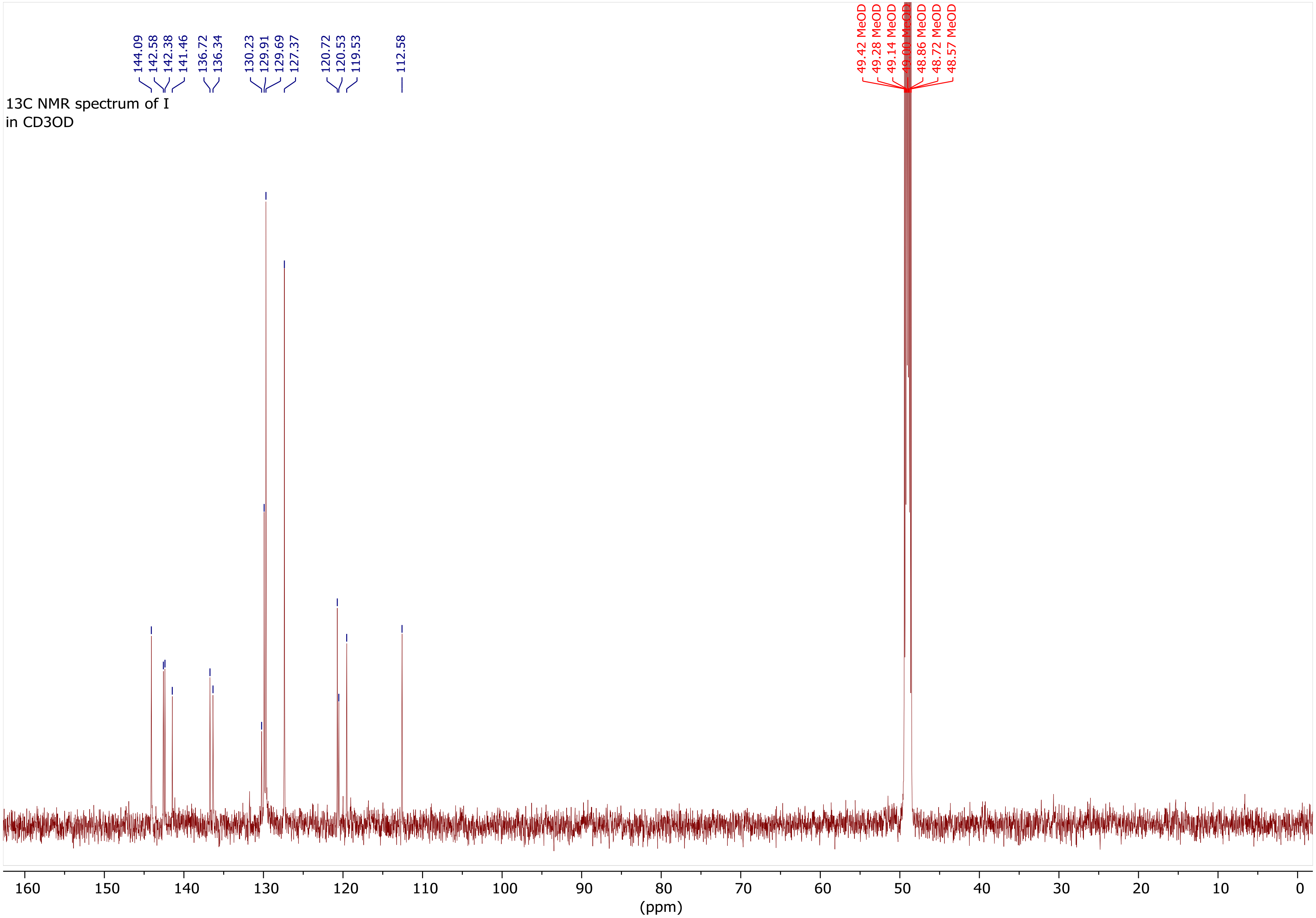

Figure S9. ^{1}H-^{13}C HSQC NMR spectrum of the compound **I** (*anti*-isomer + ~20% *syn*-isomer).

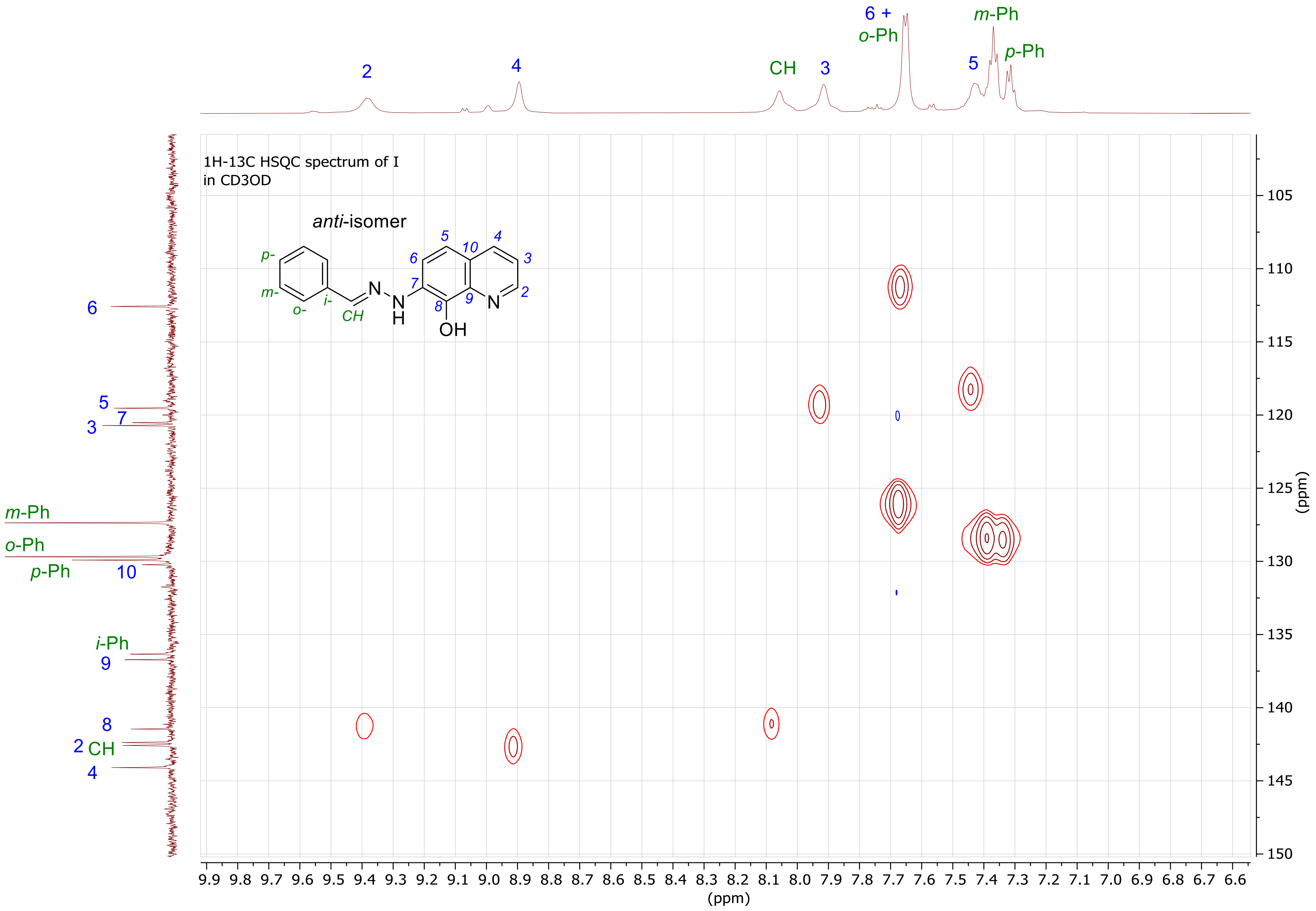

Figure S10. ^{1}H NMR spectrum of the compound **II** (*anti*-isomer + 40% *syn*-isomer).

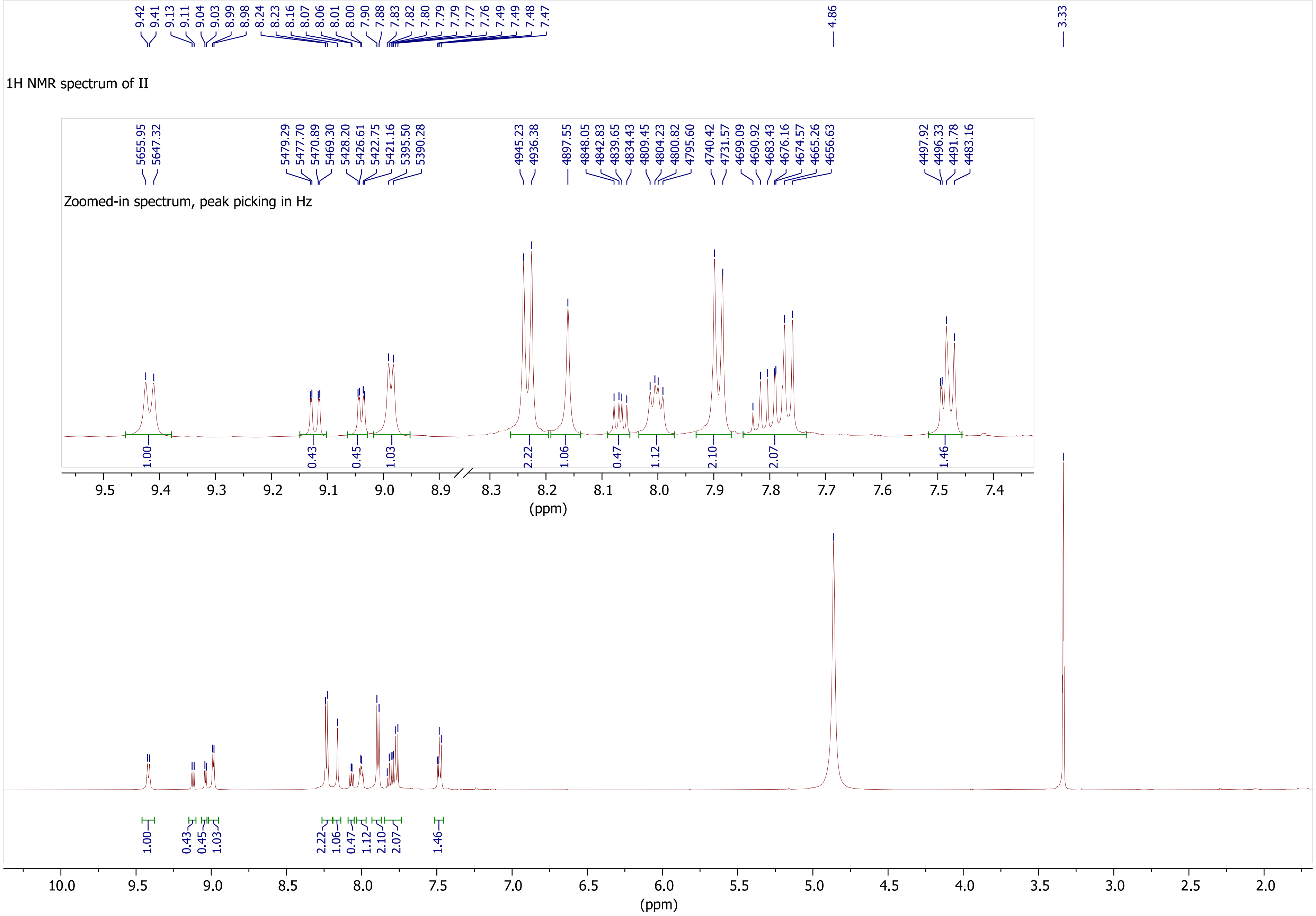

Figure S11. ^{13}C NMR spectrum of the compound **II** (*anti*-isomer + 40% *syn*-isomer).

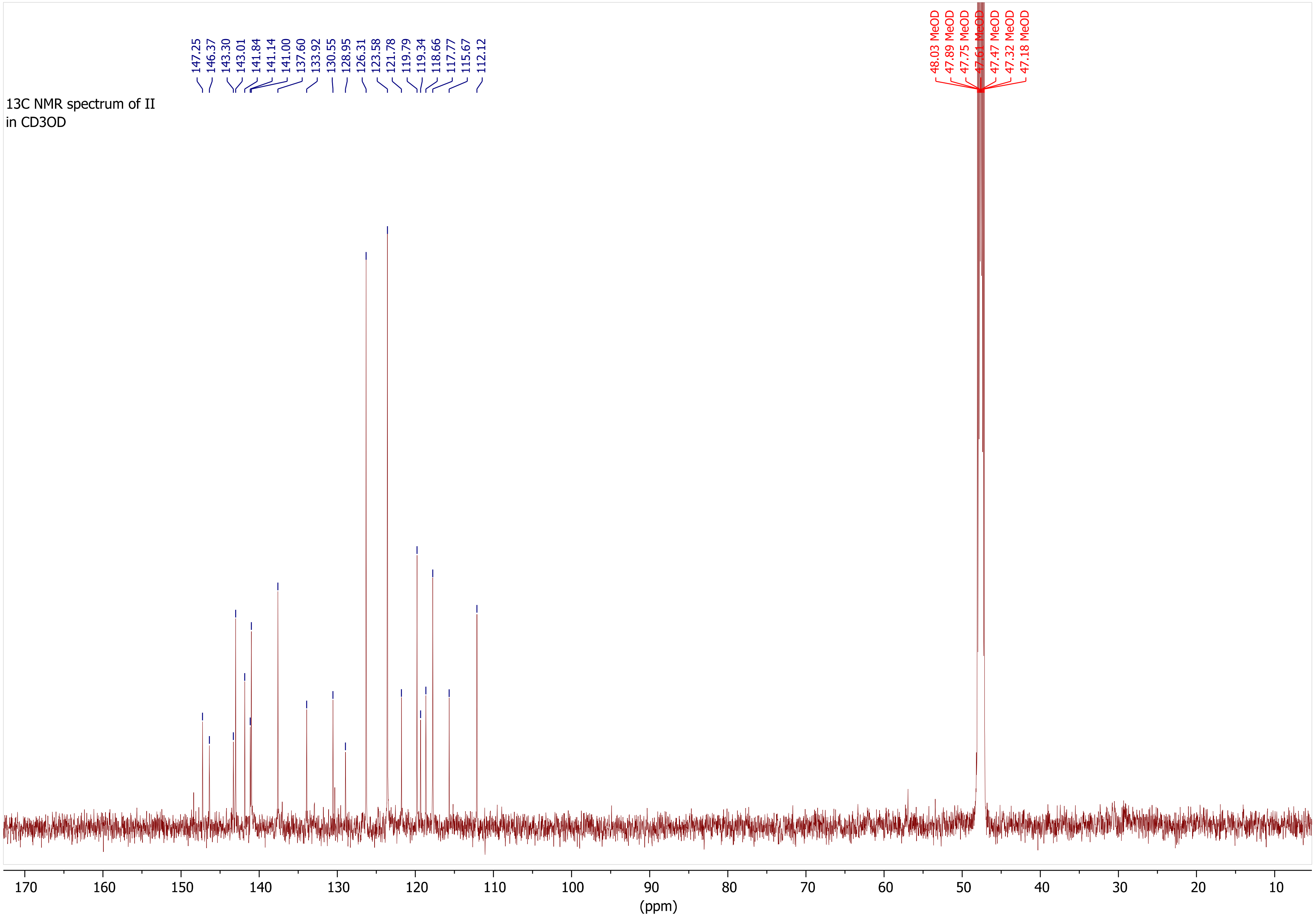

Figure S12. $^{1}H-^{13}C$ HSQC NMR spectrum of the compound **II** (*anti*-isomer + 40% *syn*-isomer).

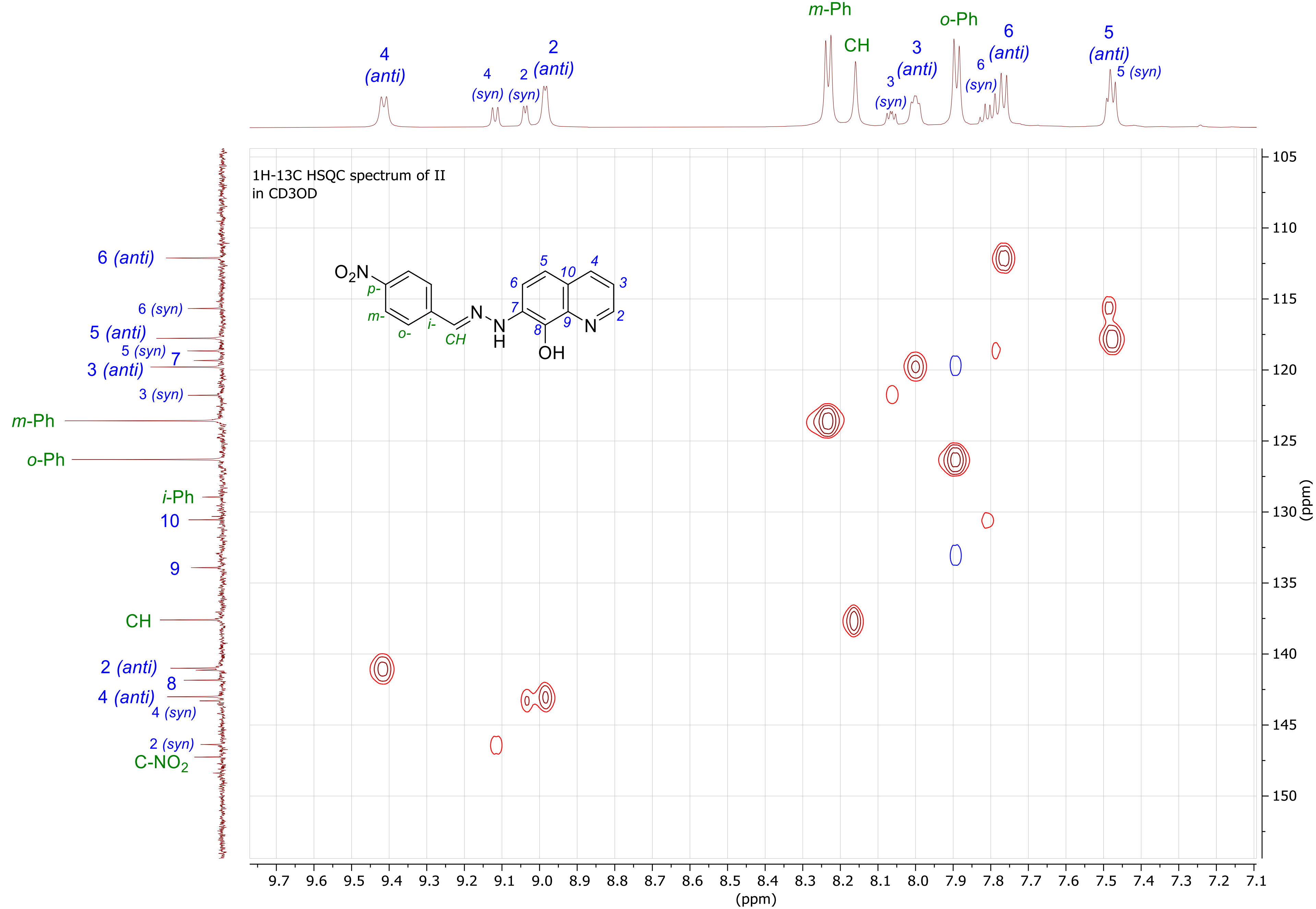

Figure S13. ^{1}H NMR spectrum of the compound **III** (*anti*-isomer + 10% *syn*-isomer).

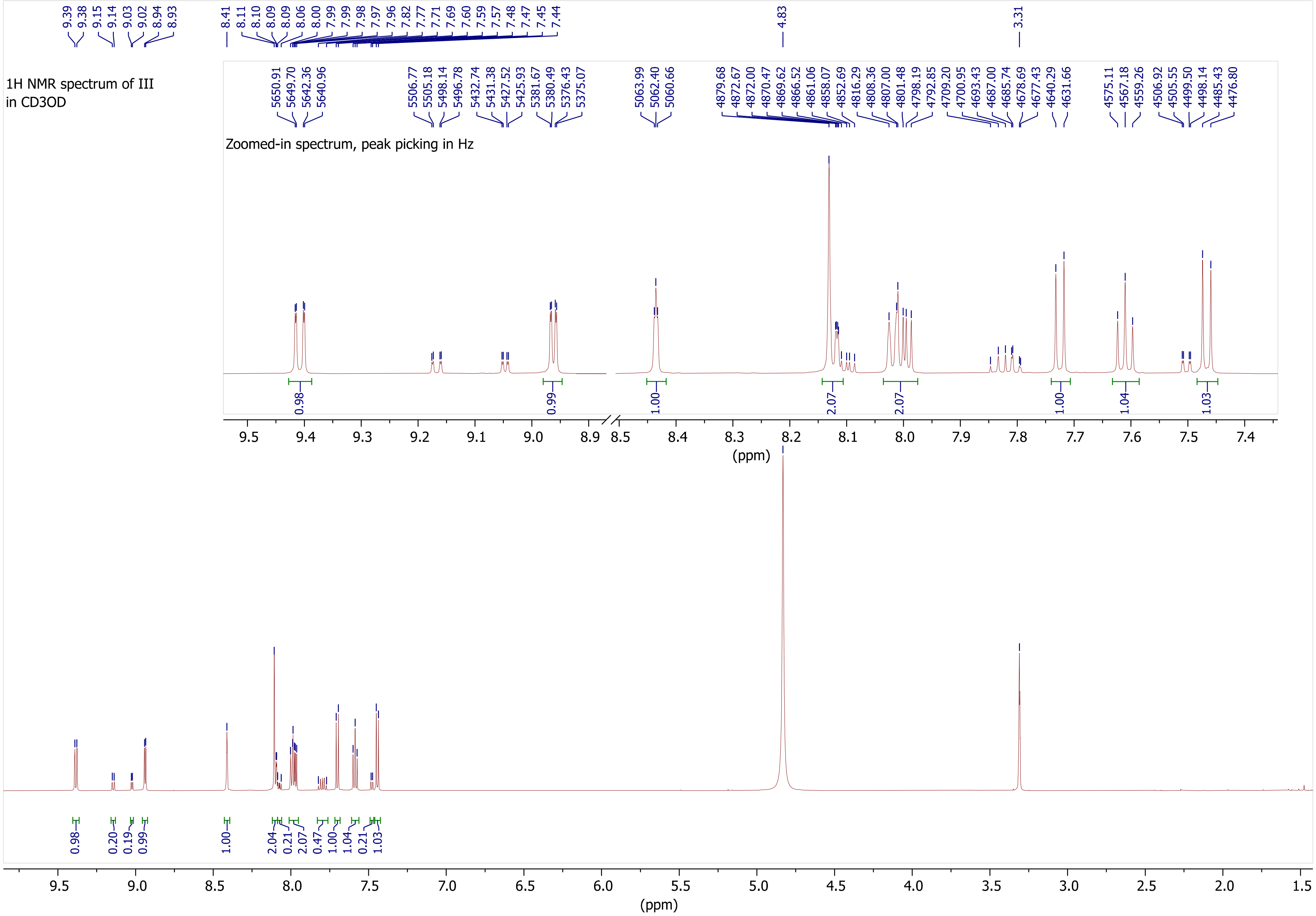

Figure S14. ^{13}C NMR spectrum of the compound **III** (*anti*-isomer + 10% *syn*-isomer).

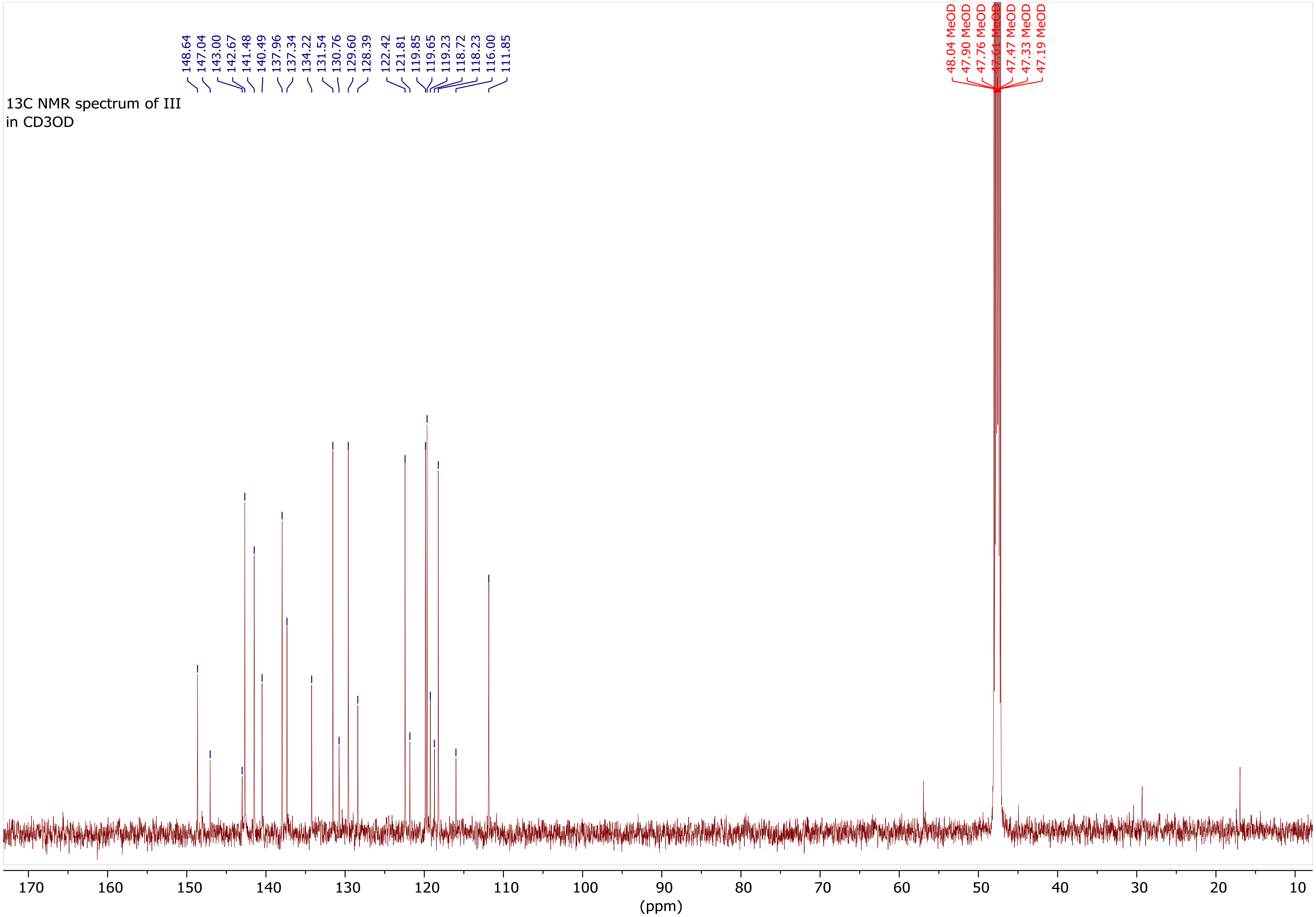

Figure S15. ^{1}H-^{13}C HSQC NMR spectrum of the compound **III** (*anti*-isomer + 10% *syn*-isomer).

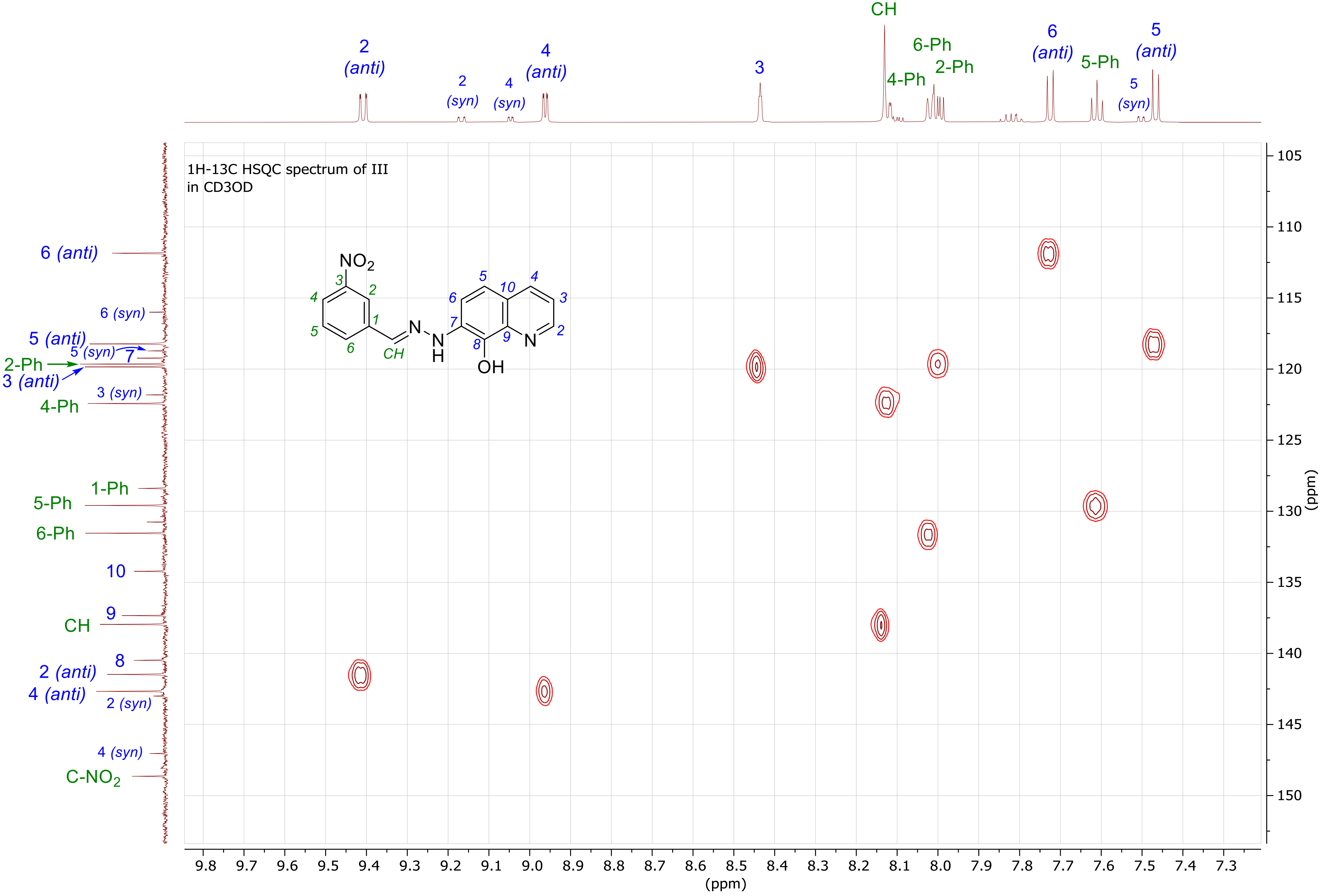

Figure S16. ^{1}H NMR spectrum of the compound **IV** (*anti*-isomer + 10% *syn*-isomer).

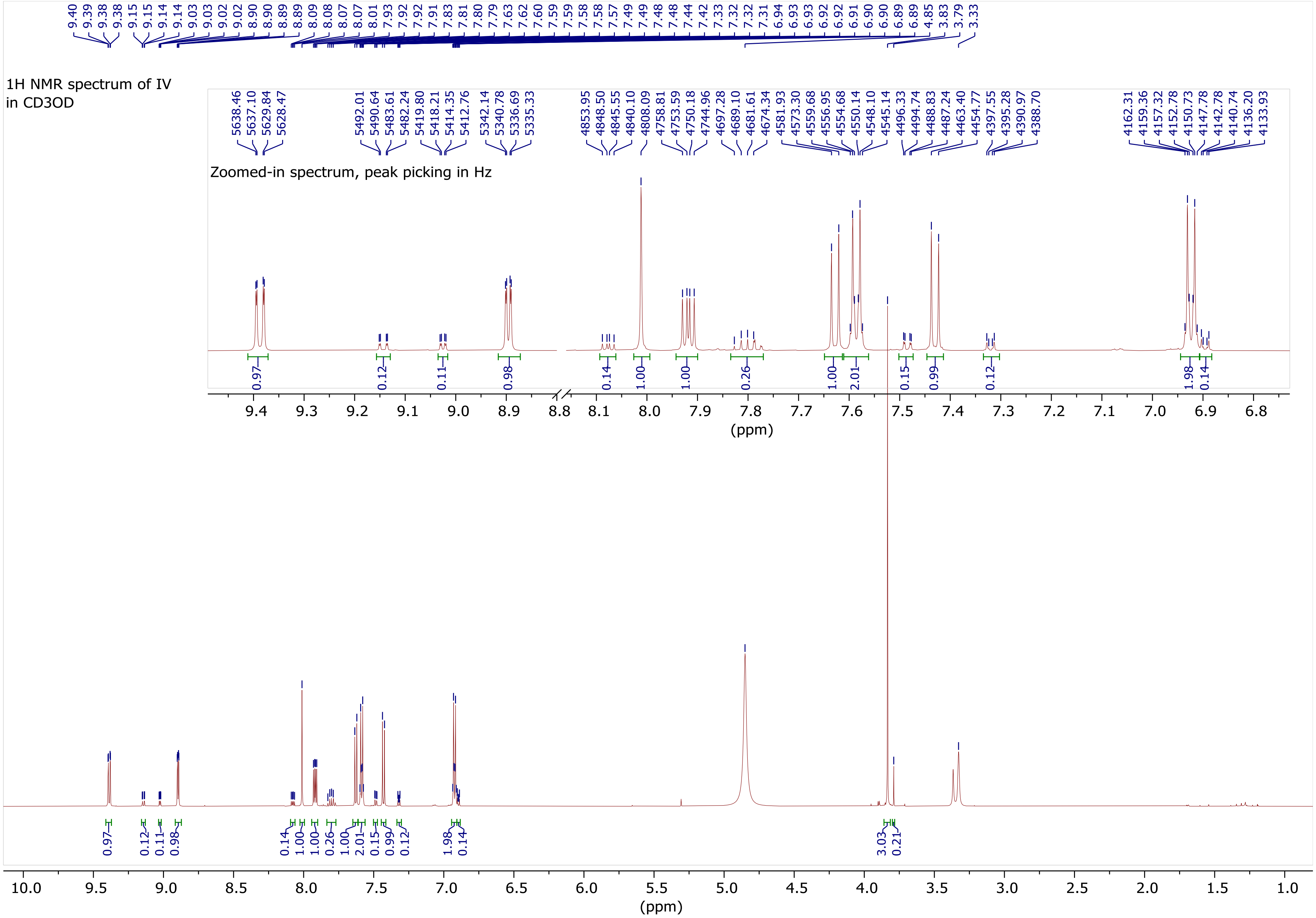

Figure S17. ^{13}C NMR spectrum of the compound **IV** (*anti*-isomer + 10% *syn*-isomer).

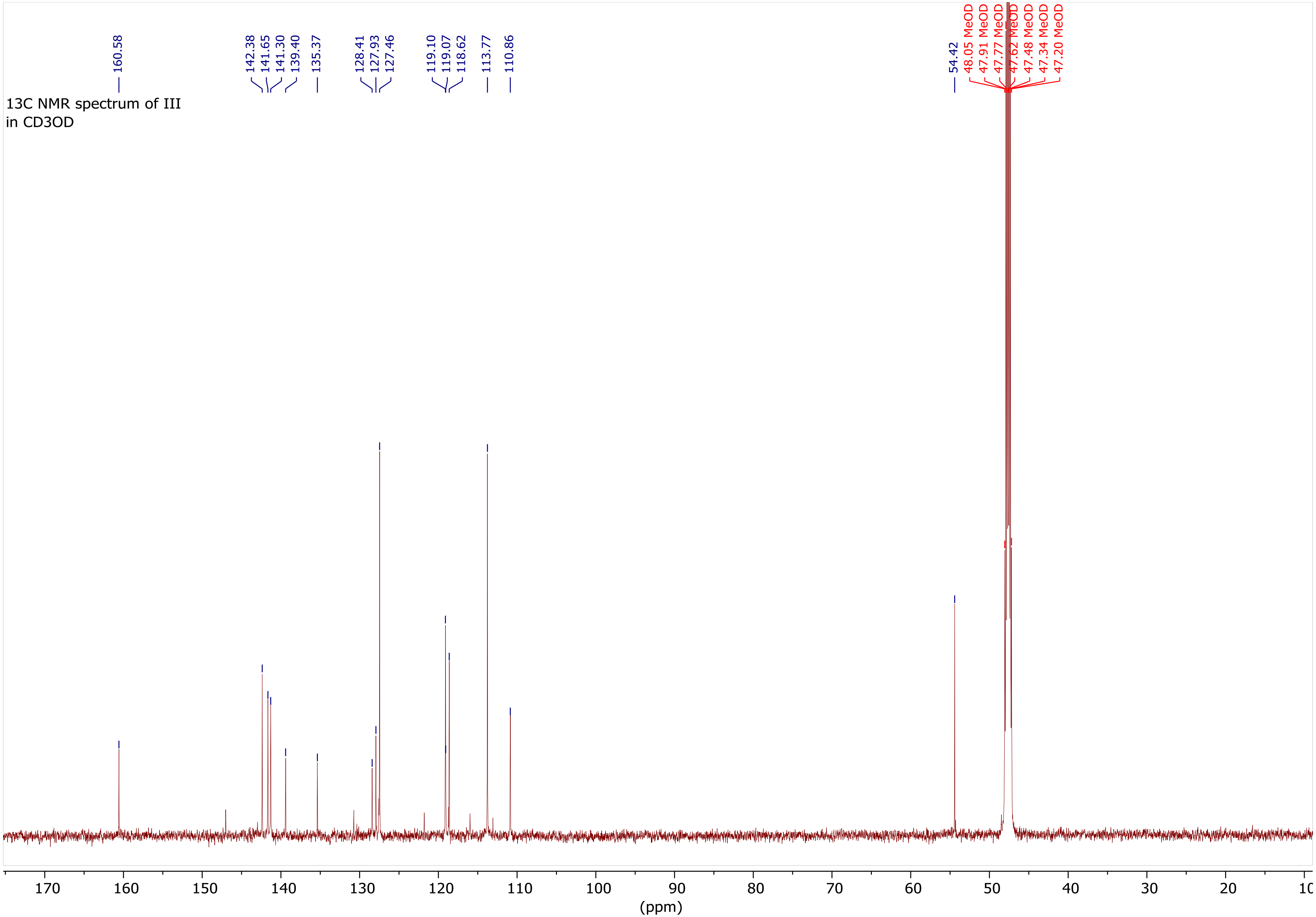

Figure S18. ^{1}H-^{13}C HSQC NMR spectrum of the compound **IV** (*anti*-isomer + 10% *syn*-isomer).

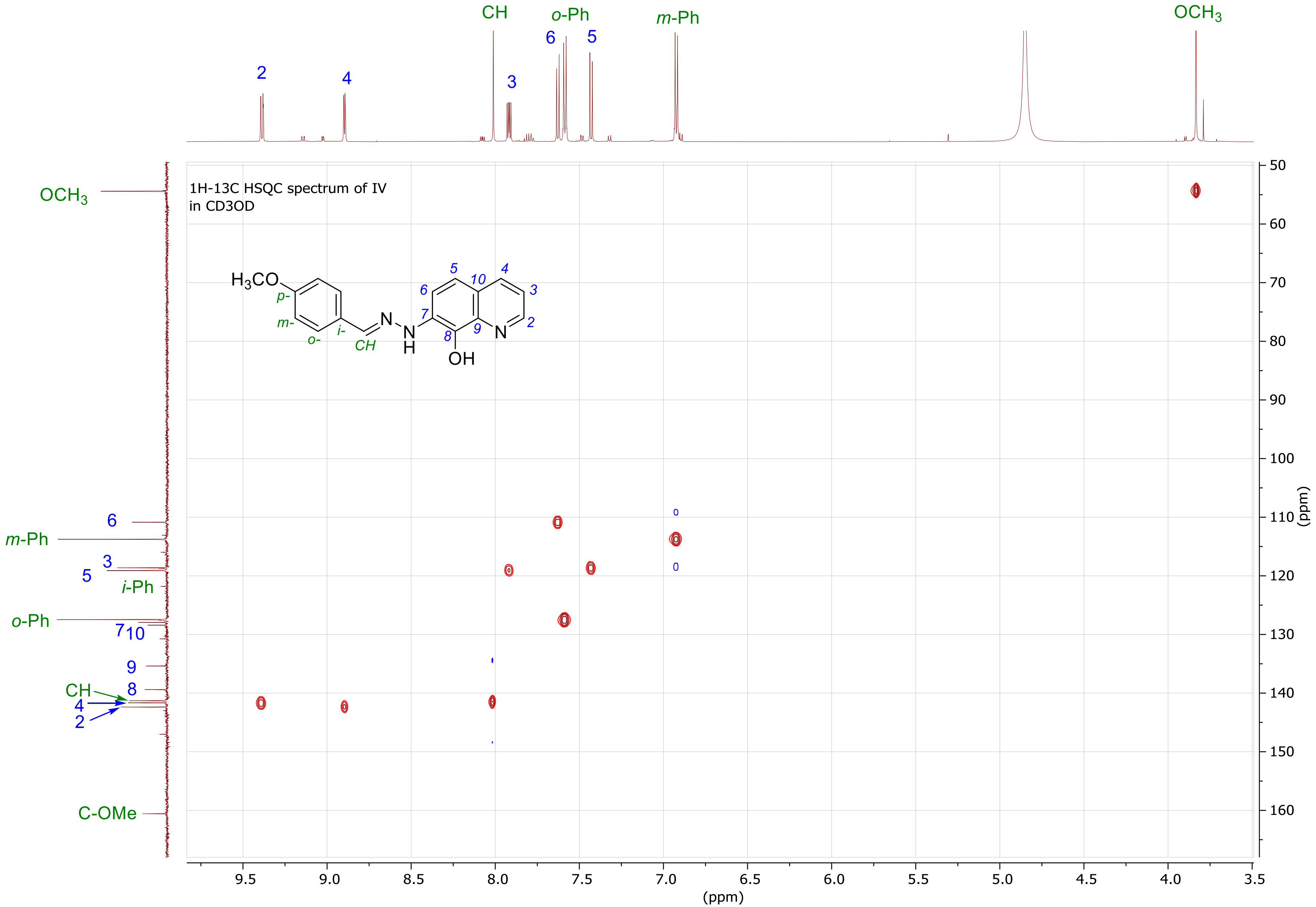

Figure S19. ^{1}H NMR spectrum of the compound **V** (*anti*-isomer + 10% *syn*-isomer).

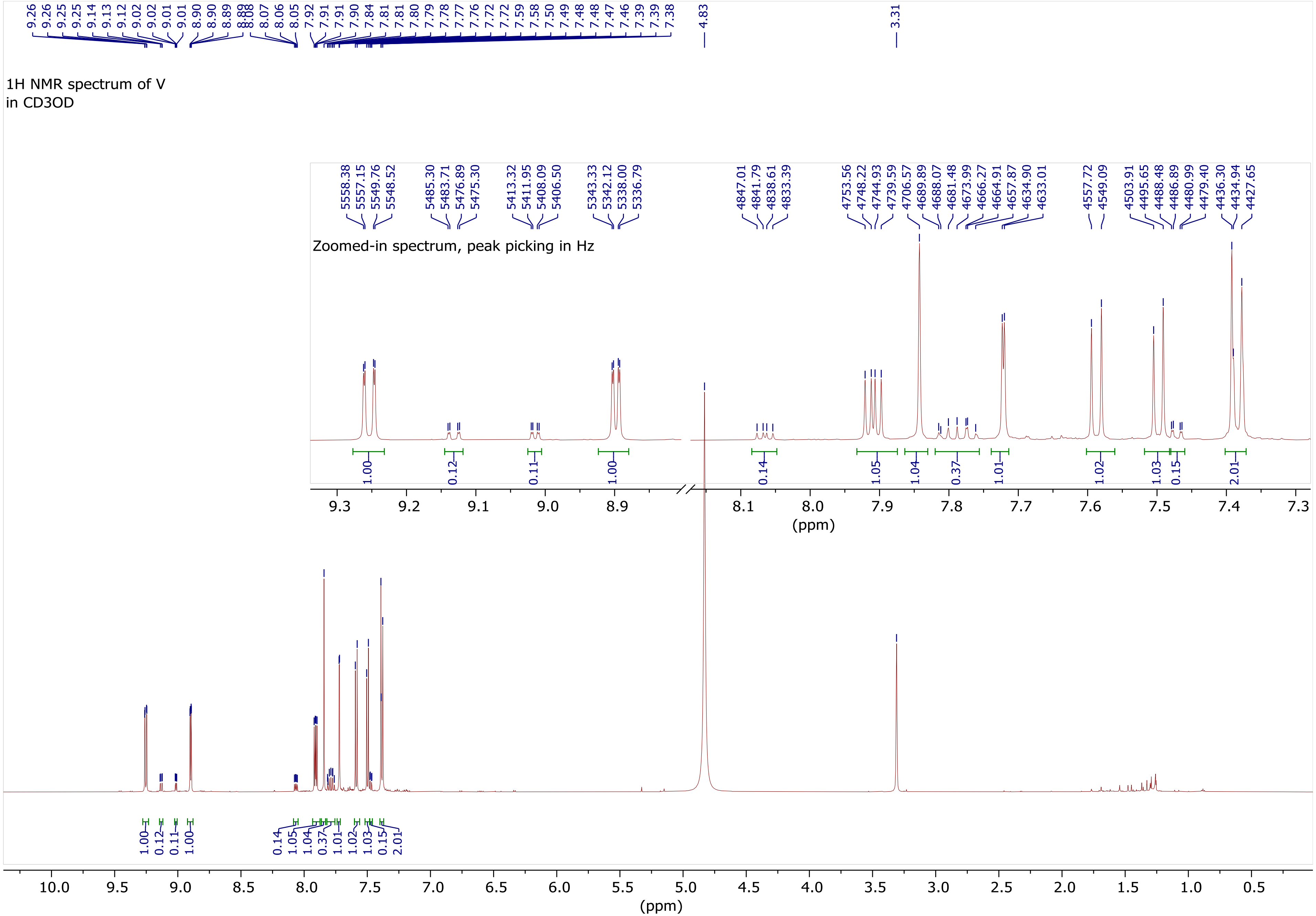

Figure S20. ^{13}C NMR spectrum of the compound **V** (*anti*-isomer + 10% *syn*-isomer)..

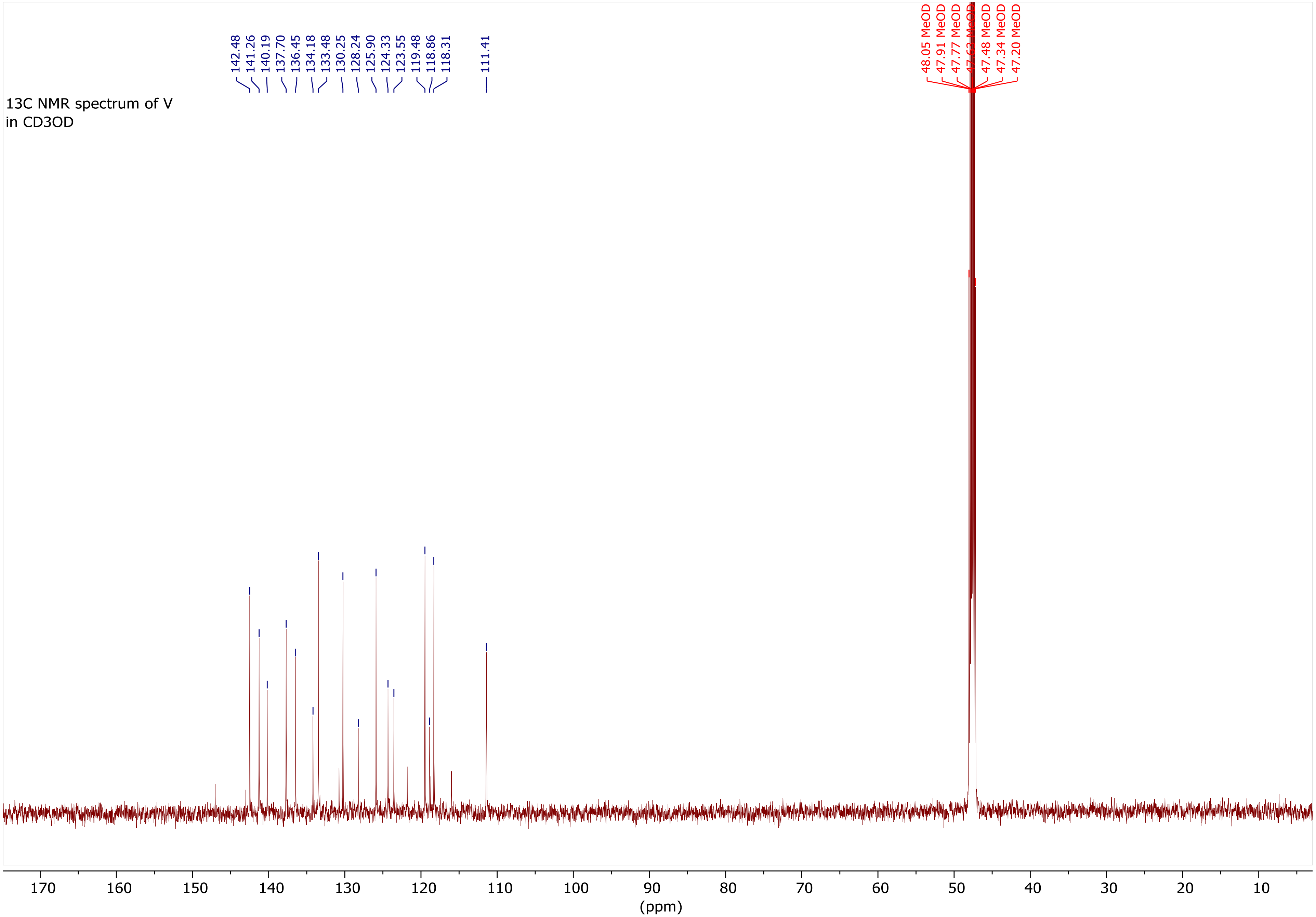

Figure S21. ^{1}H-^{13}C HSQC NMR spectrum of the compound **V** (*anti*-isomer + 10% *syn*-isomer).

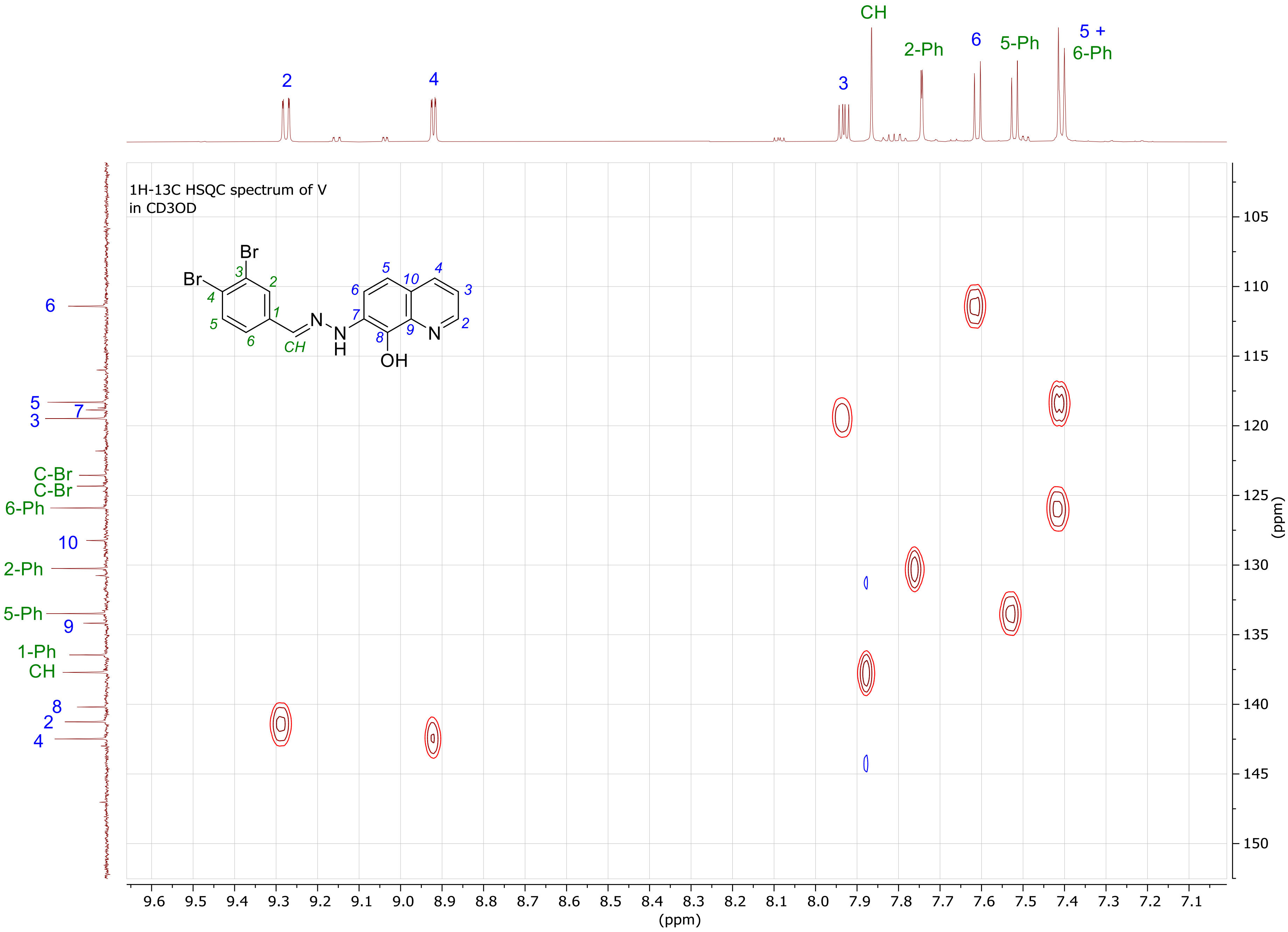

Figure S22. ^{1}H NMR spectrum of the compound **VI** (*anti*-isomer + ~15% *syn*-isomer).

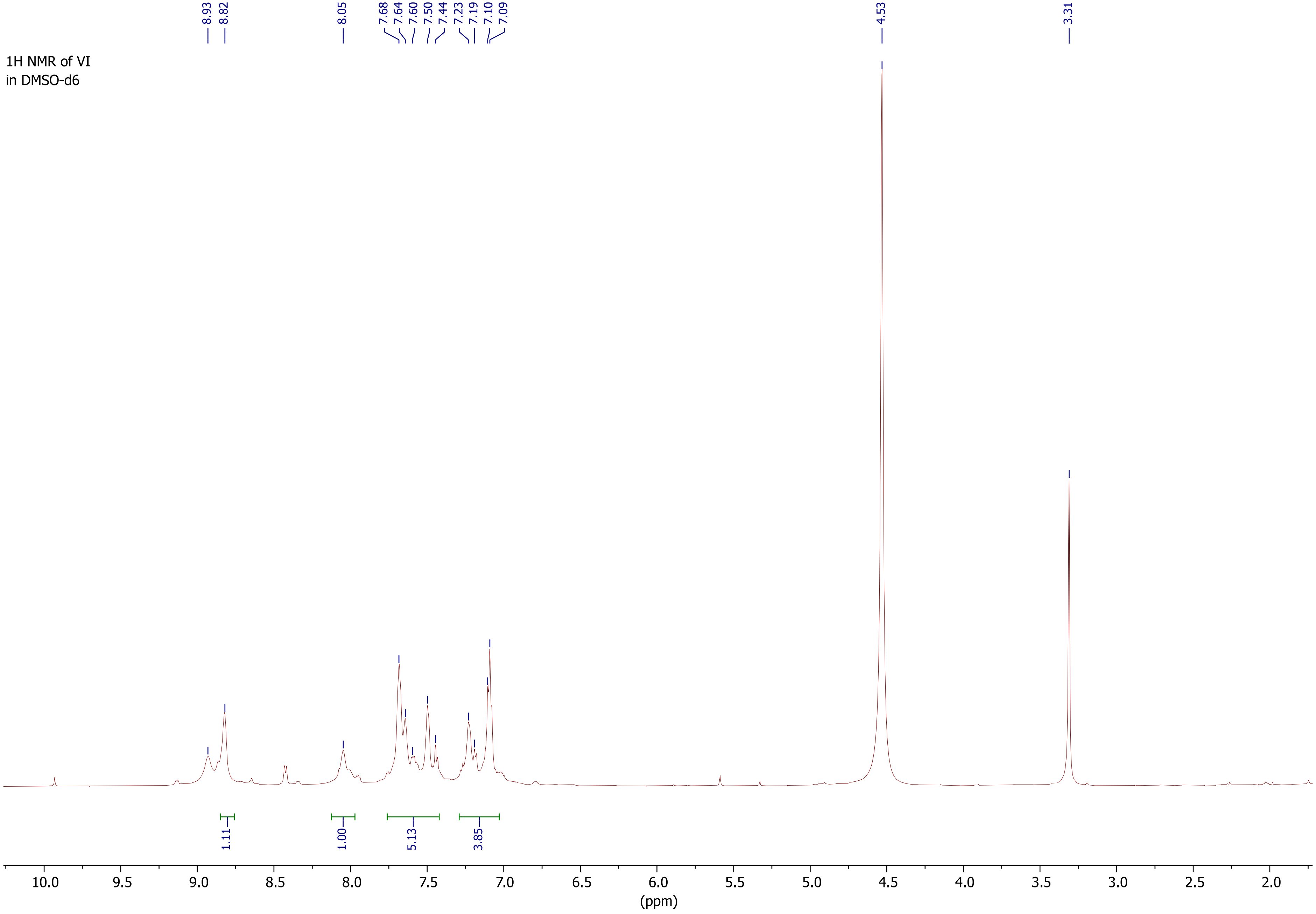

Figure S23. ^{13}C NMR spectrum of the compound **VI** (*anti*-isomer + ~15% *syn*-isomer).

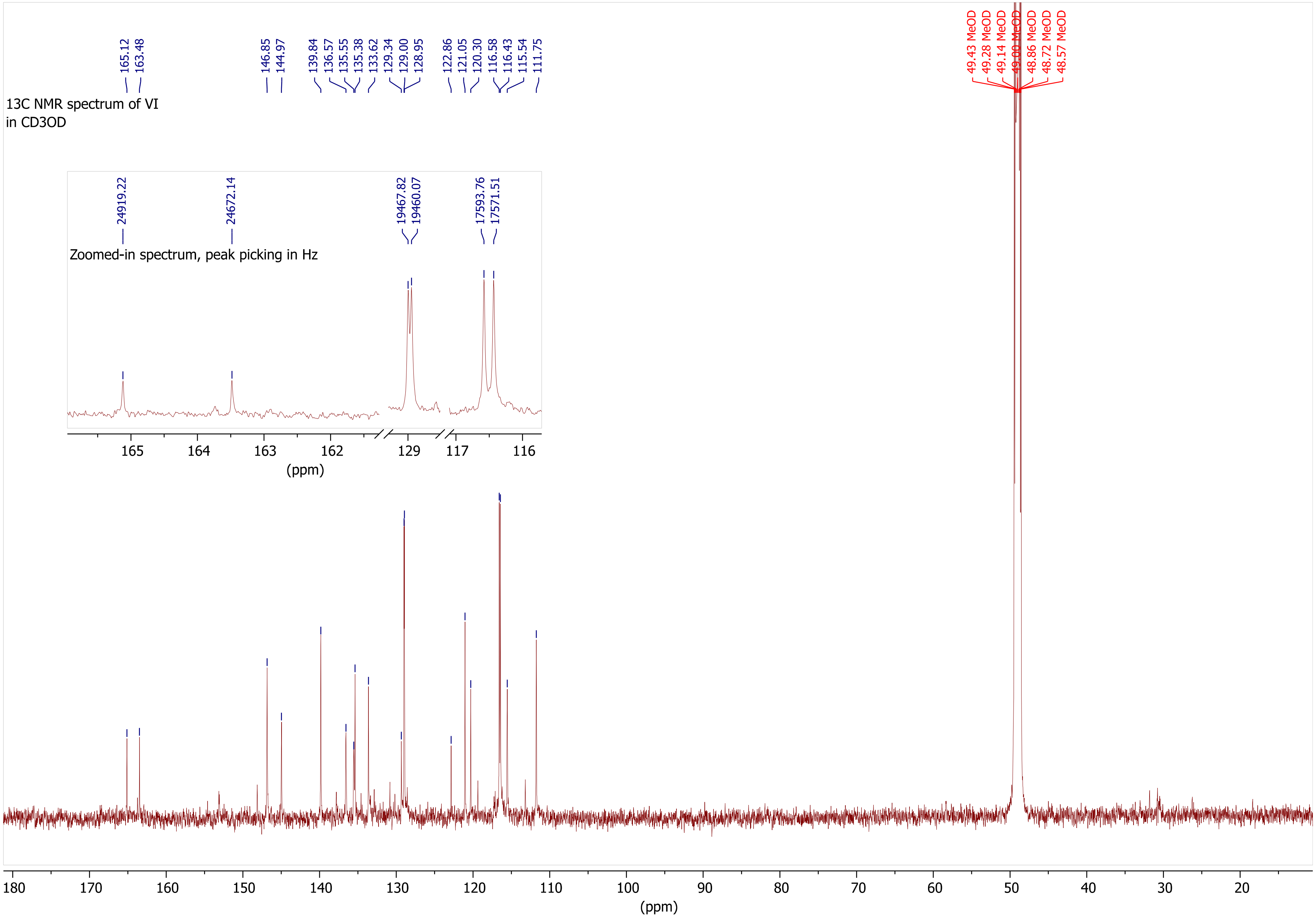

Figure S24. ^{1}H-^{13}C HSQC NMR spectrum of the compound **VI** (*anti*-isomer + ~15% *syn*-isomer).

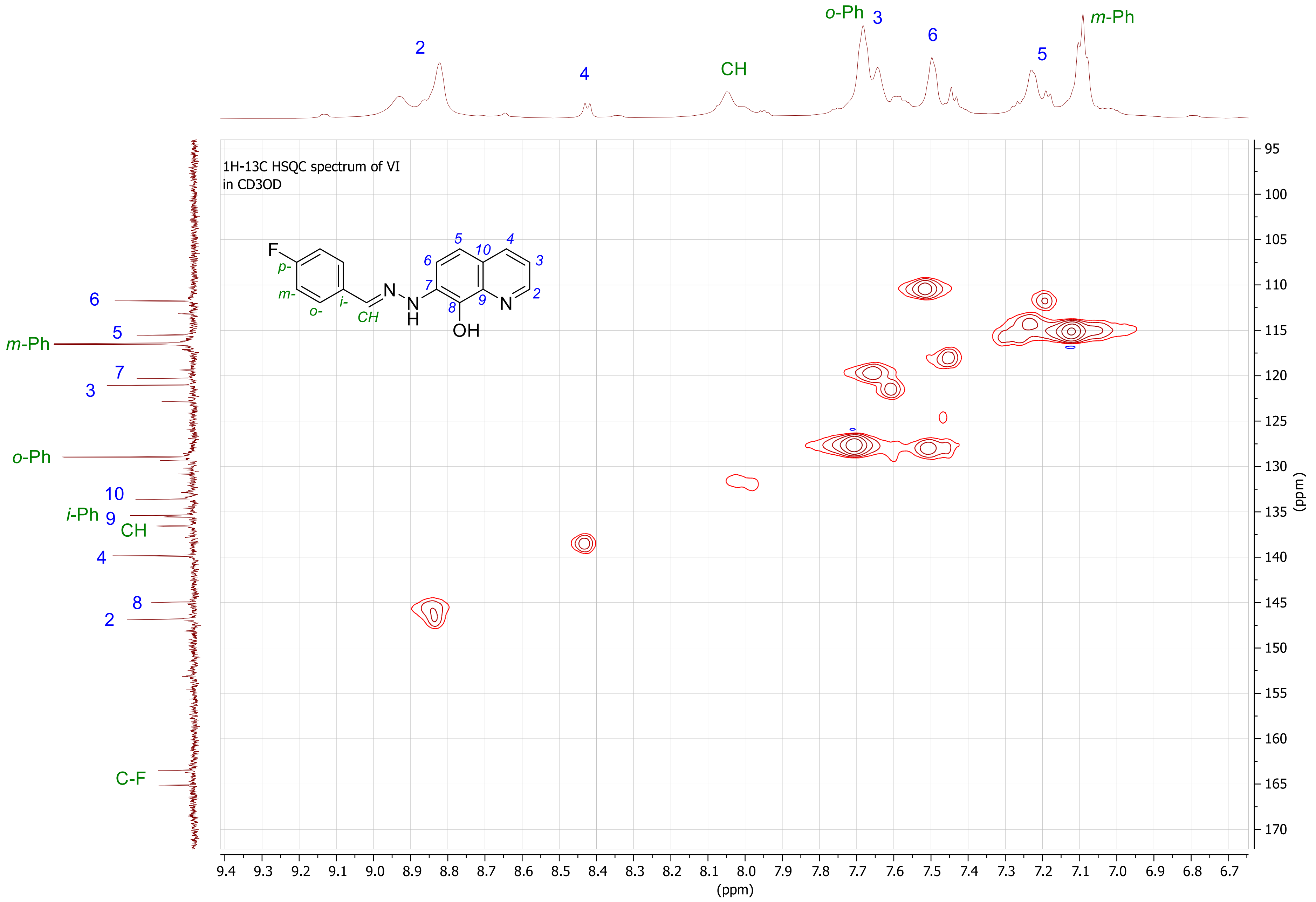

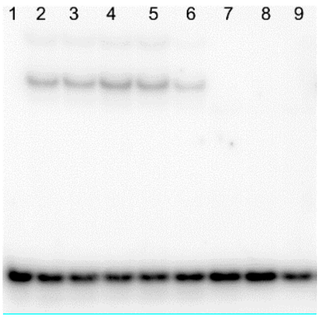

Figure S25. EMSA analysis of the Ku/DNA interaction inhibition by **V**. 1 – negative control in the absence of Ku, 2 – positive control of DNA binding in the absence of inhibitor, 3-9 – binding with the increasing concentration of the inhibitor: 3.9; 7.8; 15.6; 31.3; 62.5; 125 and 250 μM, respectively. Gels were transferred onto a Whatman paper, lyophilized and visualized as a radioautogram (see Materials and Methods section)

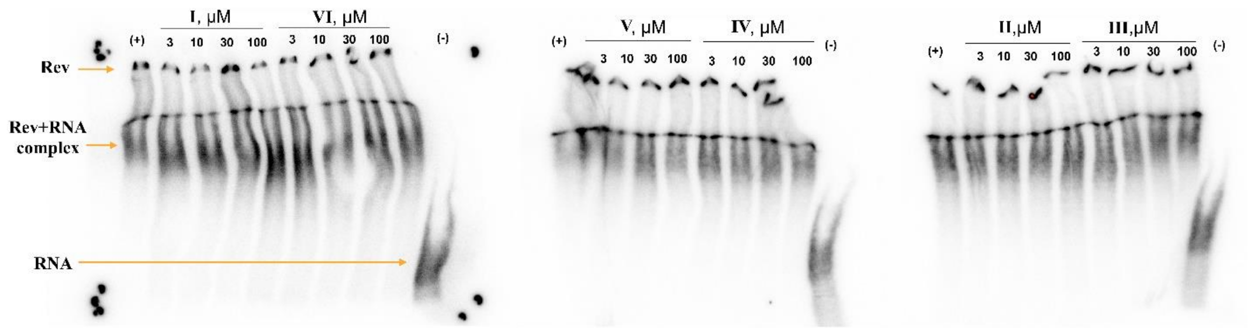

Figure S26. EMSA analysis of the Rev–RNA complex interaction with hydrazones **I-VI**. Radioactive labeled RNA (RNA*) was incubated with the compounds and Rev protein ("+ and – refer to the presence or absence of Rev in the reaction); The protein, RNA and the compounds were incubated at +4°C for 1 h in RBB buffer, then loading buffer was added and the mixture was subjected to native 4% SDS–PAGE. Gels were transferred onto a Whatman paper (images are represented with the slightly warped distortion due to the laborious transfer of the frail gel), lyophilized and visualized as a radioautogram (other details in Materials and Methods section)

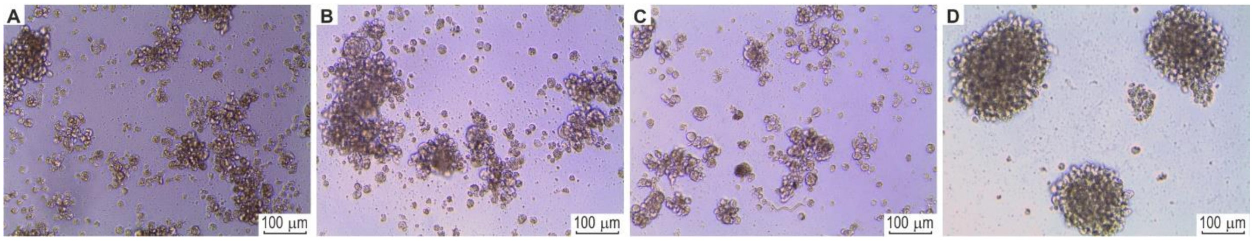

Figure S27. Effects of compound **VI** and EFV on the syncytia formation (in 3 days after infection). (A) infected MT-4 cell culture growing in the presence of **VI** (5 μM); (B) infected MT-4 cell culture growing in the presence of **VI** (1 μM); (C) infected MT-4 cell culture growing in the presence of **VI** (0.5 μM); (D) infected cell culture growing in the presence of EFV (0.5 μM).

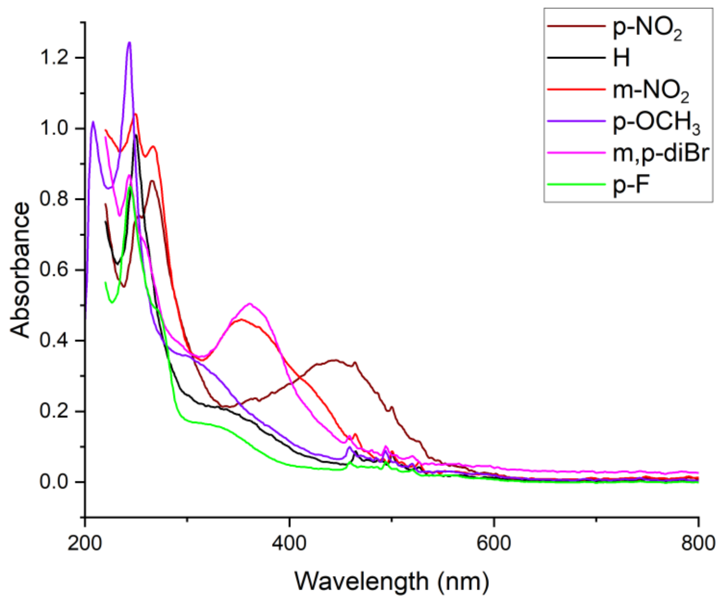

Figure S28. UV-Vis absorption spectra of 7-benzylidenohydrazinyl-8-hydroxyquinolines

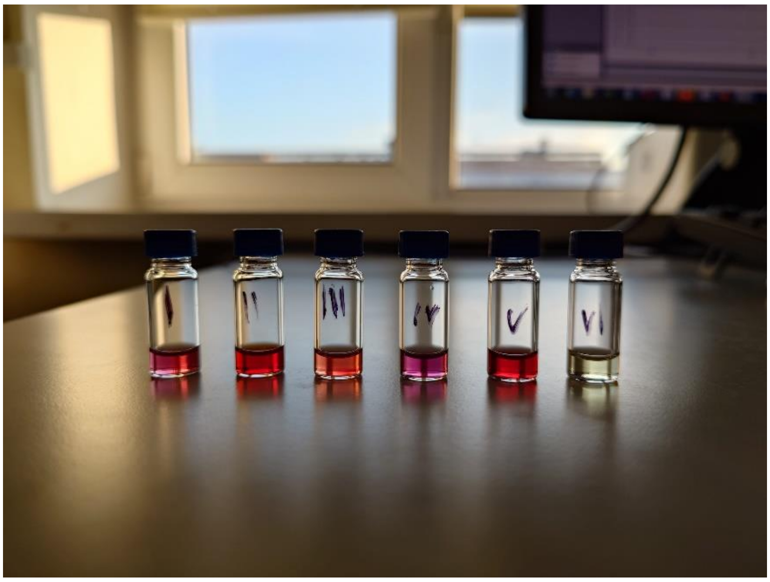

Figure S29. 7-Benzylidenohydrazinyl-8-hydroxyquinolines in diluted methanol solutions

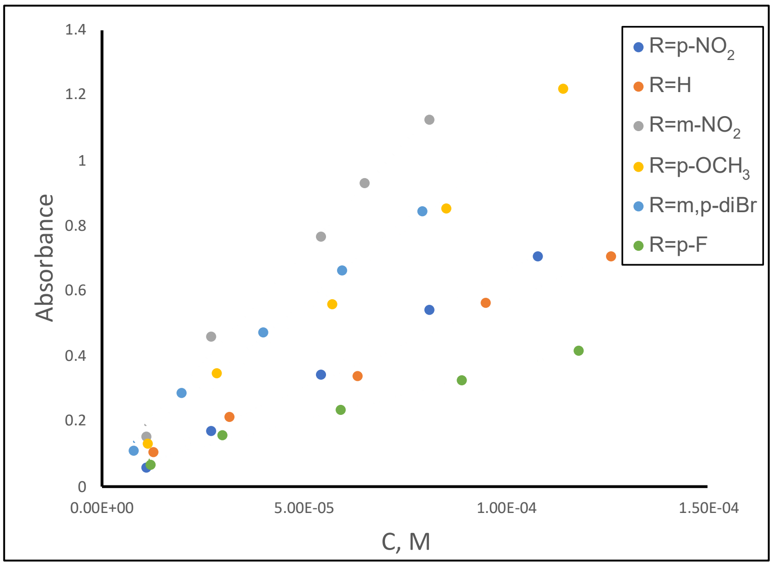

Figure S30. Calibration curves of the compounds at the selected λ_{max}

Table S1. Calculated data for hydrazone-incorporated microgel formation

**Compound**	**λ_{max}, nm**	**Absorbance exp., Abs**	**Absorbance theor., Abs**	**m exp., μg**	**Load exp., % wt.**	**Load theor., % wt.**
R=*p*-NO_2	450	0.50	0.52	229.80	28.73	33.00
R=H	330	0.43	0.50	228.98	28.61	33.00
R=*m*-NO_2	354	0.83	1.07	209.03	26.12	33.00
R=*p*-OCH_3	335	0.78	0.92	205.63	25.75	33.00
R=*m,p*-diBr	375	0.60	0.63	250.19	31.27	33.00
R=*p*-F	333	0.23	0.30	198.45	24.81	33.00

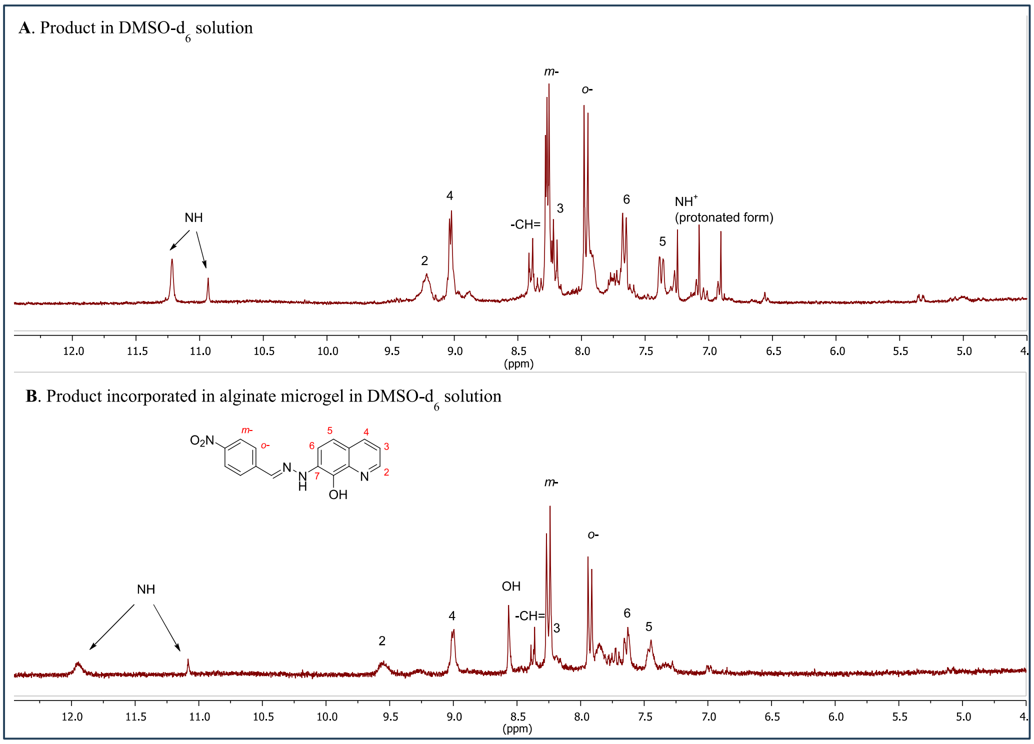

Figure S31. ^{1}H-NMR spectrum of the hydrazone **II** (a mixture of tautomeric forms in DMSO-d_6) (A) and the sample of **II** incorporated into the alginate microgel (B).

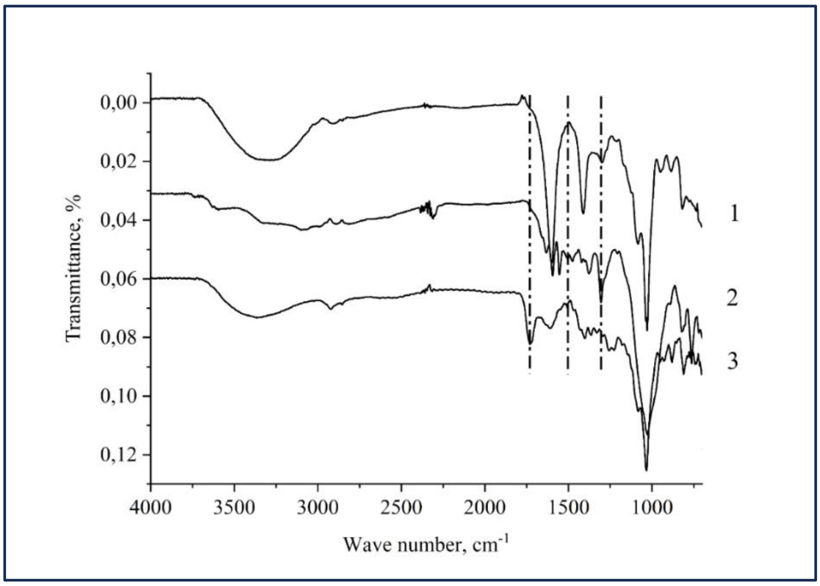

Figure S32. IR spectra of the alginate microgel without the included substance (1), of the hydrazone **II** (R=*p*-NO_2) (2), and of the microgel with the incorporated hydrazone **II** (3).
